# Navigation Systems Significantly Improve the Efficiency and Safety of CT-Guided Interventions

**DOI:** 10.3390/life16030431

**Published:** 2026-03-06

**Authors:** Mátyás Rédei, Petra Sólymos, Caner Turan, Bence Szabó, Alexandra Ádám, Ioana-Irina Rezuș, Zsolt Molnár, Gábor Duray, Péter Hegyi, Dénes Balázs Horváthy

**Affiliations:** 1 Centre for Translational Medicine, Semmelweis University, 1085 Budapest, Hungary; redei.matyas@semmelweis.hu (M.R.); solymos.petra@semmelweis.hu (P.S.); caner.turan@semmelweis.hu (C.T.); szabo.bence1@semmelweis.hu (B.S.); adam.alexandra@stud.semmelweis.hu (A.Á.); rezusioanairina@gmail.com (I.-I.R.); molnar.zsolt1@semmelweis.hu (Z.M.); gduray@yahoo.com (G.D.); peter.hegyi@semmelweis.hu (P.H.); 2 Department of Radiology, Medical Imaging Centre, Semmelweis University, 1085 Budapest, Hungary; 3 Department of Anesthesiology and Intensive Therapy, Semmelweis University, 1085 Budapest, Hungary; 4 Institute for Translational Medicine, Medical School, University of Pécs, 7622 Pécs, Hungary; 5 Department of Radiology, University of Medicine and Pharmacy “Grigore T. Popa”, 700115 Iasi, Romania; 6 Radiology Clinic, “Sfantul Spiridon” County Clinical Emergency Hospital, 700111 Iasi, Romania; 7 Department of Anesthesiology and Intensive Therapy, Poznan University of Medical Sciences, 61-701 Poznan, Poland; 8 Department of Cardiology, Central Hospital of Northern Pest—Military Hospital, 1134 Budapest, Hungary; 9 Heart and Vascular Centre, Semmelweis University, 1085 Budapest, Hungary; 10 Institute of Pancreatic Diseases, Semmelweis University, 1085 Budapest, Hungary; 11 Department of Interventional Radiology, Heart and Vascular Centre, Semmelweis University, 1085 Budapest, Hungary

**Keywords:** CT-guided intervention, navigation system, image-guided biopsy, ablation, radiation dose, meta-analysis

## Abstract

**Objectives**: CT-guided interventions are associated with radiation exposure, prolonged procedural time, and complications. Navigation systems (NS) have been developed to improve procedural precision and efficiency. This study aimed to evaluate the impact of NS on procedural outcomes, radiation dose, and complication rates compared with conventional freehand techniques. **Materials and methods:** A systematic review and meta-analysis was performed including 30 studies (11 randomized controlled trials, 19 cohort studies) published through November 2023, involving 2785 patients (1418 NS; 1367 control). Outcomes included the number of needle manipulations, procedural time, radiation dose, complication rates, technical success, and diagnostic success. Random-effects models were applied with subgroup analyses by study design, intervention type, and target organ. Risk of bias was assessed using RoB 2 and ROBINS-I, and certainty of evidence using the GRADE framework. **Results:** Navigation systems significantly reduced needle manipulations (mean difference [MD], −2.58; 95% CI: −3.30 to −1.85) and procedural time (MD, −8.07 min; 95% CI: −12.27 to −3.87). Radiation dose decreased by 37% (ratio of means [ROM], 0.63; 95% CI: 0.58–0.69). Complication rates were lower overall (odds ratio [OR], 0.64), with fewer chest tube insertions during lung ablations (OR, 0.58; 95% CI: 0.39–0.86). Diagnostic success improved (OR, 1.66; 95% CI: 1.01–2.73), whereas technical success was comparable (OR, 1.41; 95% CI: 0.89–2.24). **Conclusions:** Navigation systems significantly enhance the efficiency and safety of CT-guided interventions by reducing needle manipulations, radiation exposure, and complication rates, while improving diagnostic success.

## Simple Summary

Computed tomography-guided interventions, such as biopsies and tumor ablations, are widely used to diagnose and treat cancer, but they often require multiple needle adjustments, expose patients to radiation, and may cause complications. Navigation systems have been developed to help physicians place needles more accurately during these procedures, but their overall benefit has not been fully clarified. In this study, we systematically reviewed and combined data from 30 clinical studies involving nearly 2800 patients to compare navigation-assisted procedures with conventional techniques. We found that navigation systems reduced the number of needle adjustments, shortened procedure time, lowered radiation exposure, and decreased complication rates, while improving diagnostic success. These results suggest that navigation systems can make computed tomography-guided interventions safer and more efficient, supporting their wider adoption in clinical practice and future research.

## 1. Introduction

Patients undergoing interventions guided by computed tomography (CT) are exposed to significant risks, including increased radiation dose and extended procedural time. Standard CT-guided interventional procedures include biopsy, ablation, drainage, vertebroplasty, nerve blocks, and neurolysis [1]. Although essential for diagnostic and therapeutic purposes, these procedures may lead to complications such as pneumothorax or hemorrhage.

The gold standard freehand CT-guided percutaneous interventions involve several precise steps to ensure accuracy and minimize complications. The process begins with an initial CT scan to identify the lesion and determine the safest needle path. A gantry laser or radiopaque marker marks the entry point and the operator administers local anesthesia. Subsequently, a biopsy needle or ablation probe is incrementally advanced under CT guidance. Imaging confirms and adjusts the trajectory at each step, ensuring alignment with the lesion. This systematic approach reduces tissue trauma, optimizes accuracy, and improves procedural success [2,3].

CT fluoroscopy, an alternative to conventional freehand techniques, provides real-time imaging during needle placement, allowing continuous adjustments without repeated scans. This real-time guidance has been shown to achieve superior diagnostic accuracy, with reported rates of 88–97% compared to conventional CT (OR: 0.32, *p* < 0.00001) [4,5]. However, this increased accuracy comes at the cost of significantly higher radiation exposure to operators, with mean hand doses of 0.759 mSv compared to 0.034 mSv in conventional CT-guided methods. These trade-offs, including continuous radiation exposure, limit the broader adoption of CT fluoroscopy in clinical practice.

A major challenge of conventional methods is their dependence on operator skill, which may lead to repeated needle manipulations and repositioning [6]. This may prolong procedural time and increase radiation exposure due to repeated control CT scans.

Navigation systems in interventional radiology have evolved significantly to improve the accuracy and safety of CT-guided procedures. Navigation and robotic systems can be mounted on the patient, ceiling, floor, or table and integrated with imaging systems as a plug-in add-on [7]. Utilizing technologies such as stereotactic tracking, electromagnetic (EM) tracking, laser guidance, and augmented reality (AR), these systems provide real-time guidance for precise needle placement [1,8,9,10,11]. By improving hand–eye coordination, these systems make procedures more intuitive, reduce operator variability, and minimize the need for multiple needle manipulations [12]. The number of needle manipulations is a crucial factor that directly affects complication rates, irradiation dose, and procedural time, all of which contribute to these procedures’ overall efficacy and safety.

Despite the benefits of navigation systems, their widespread clinical adoption has been slow, primarily due to their high cost and the challenges of integrating them into existing workflows. However, robust comparative data demonstrating their benefits over conventional methods could significantly facilitate their adoption in clinical practice.

The objective of this systematic review and meta-analysis was to quantify the impact of CT-guided navigation systems compared with conventional freehand techniques on the number of needle manipulations, radiation dose, procedural time, complication rates, and technical and diagnostic success. Subgroup analyses were planned according to target organ, intervention type, and study design.

## 2. Materials and Methods

### 2.1. Study Protocol

The study protocol was prospectively registered on PROSPERO (registration ID CRD42023468539) and was adhered to throughout the study. The only deviation was to replace the “time until successful placement” outcome with “total procedural time” to align with the reporting format of the included articles. Procedural time was defined as the duration of the intervention itself, rather than total door-to-door time.

### 2.2. Eligibility Criteria

Applying the PICO (Population, Intervention, Comparator, Outcome) framework, we included randomized control trials (RCT), non-randomized controlled trials, and observational analytical trials where P: patients undergoing CT-guided interventions; I: utilization of navigation systems; C: conventional CT-guided method; O: The primary outcome was the number of needle manipulations. Secondary outcomes included irradiation dose, procedural time, complication rates, and technical and diagnostic success. We excluded articles from the study that utilized alternative radiological modalities, such as ultrasound or magnetic resonance imaging. In addition, case reports, case series, and conference papers were excluded from the analysis.

### 2.3. Information Sources and Search Strategy

A systematic search was performed across four major databases—PubMed, EMBASE, Cochrane, and Scopus—on 14 November 2023, using the same search key across all platforms. The Supplemental Material (Supplemental Table S1) provides the detailed search strategy. Additionally, we conducted a citation chase for references from related articles.

### 2.4. Selection Progress and Data Extraction

In two independent groups, four authors (M.R., P.S., I.I.R., and A.A.) conducted the selection process using reference management software (EndNote 20 [13]). After automatic and manual removal of duplicates, titles and abstracts were screened, followed by full-text screening according to predefined eligibility criteria. Agreement between reviewers was calculated using the Cohen κ coefficient. Using a standardized data extraction sheet, four authors in two independent groups performed data extraction (M.R., P.S., I.I.R., and A.A.). Additional data extraction included digital object identifier, first author, publication year, countries, centers, study period, study population characteristics (age, gender), intervention type, and targeted organ.

### 2.5. Subgroup Analysis

Subgroup analysis was pre-designed to stratify data by targeted organ (liver or lung), procedural type (ablation or biopsy), and study design (RCT, cohort, or non-randomized prospective study).

### 2.6. Risk of Bias Assessment and Evidence Level

Three authors (M.R., I.I.R., and A.A.) independently performed the risk of bias assessment using the revised Cochrane risk-of-bias tool for randomized trials (RoB 2) and Risk Of Bias In Non-randomized Studies—of Interventions (ROBINS-I) and GRADEpro GDT (McMaster University and Evidence Prime, Hamilton, ON, Canada) to assess the quality of evidence, with disagreements resolved by another author (C.T.).

### 2.7. Synthesis Method

We required a minimum of three studies for inclusion in the meta-analysis. Anticipating significant heterogeneity between studies, we employed a random-effects model to aggregate effect sizes. The number of needle manipulations was considered the primary outcome, as it represents the key procedural factor influencing radiation exposure, procedural time, and complication risk.

The effect size was measured using the odds ratio (OR) with a corresponding 95% confidence interval (CI) for dichotomous outcomes. For continuous outcomes, the effect size was assessed either by the difference between means (MD) or, in cases where outcomes were reported in different units, the ratio of means (ROM) was utilized. We extracted the sample size, mean, and standard deviation (SD) from each study to calculate the pooled difference. In cases where the median and interquartile range (IQR) were reported, we computed the mean and SD accordingly.

We employed a random intercept logistic regression model to pool proportions. To calculate pooled OR based on raw data, we utilized the Mantel–Haenszel method. The exact Mantel–Haenszel method (without continuity correction) was applied in cases of zero cell counts. Results were deemed statistically significant if the confidence interval (CI) did not include the null value.

Findings were summarized using forest plots with prediction intervals. Heterogeneity was assessed using Higgins and Thompson I^2^ statistics. All statistical analyses were performed using the R software version 3.6.1 (R Foundation for Statistical Computing, Vienna, Austria) [14].

If needed and possible, model fitting parameters and potential outlier publications were explored using different influence measures and plots (e.g., leave-one-out analysis for changes in fitted values, Bujat diagnostic values, and corresponding plots). Small-study publication bias was assessed by visual inspection of funnel plots and Egger’s test at a 10% significance level.

### 2.8. Reporting Standards

This systematic review and meta-analysis was conducted in accordance with the PRISMA 2020 (Preferred Reporting Items for Systematic Reviews and Meta-Analyses) guidelines. The completed PRISMA 2020 checklist is provided in the Supplemental Material (Supplemental Table S2).

## 3. Results

### 3.1. Study Selection and Characteristics

**Figure 1:**
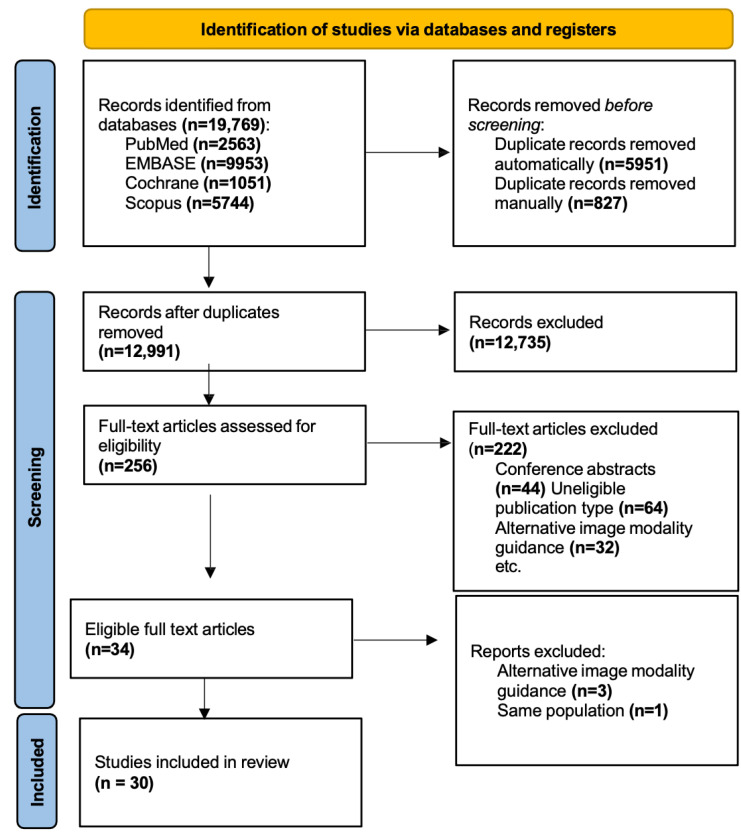
PRISMA flowchart of selection.

Our systematic search across PubMed, EMBASE, Cochrane, and Scopus databases on 14 November 2023 yielded 12,991 records after duplicate removal. A total of 256 studies were eligible based on titles and abstracts, and 36 full-text articles met our predefined eligibility criteria. Our final meta-analysis included data from 30 studies encompassing RCTs and cohort studies [9,15,16,17,18,19,20,21,22,23,24,25,26,27,28,29,30,31,32,33,34,35,36,37,38,39,40,41,42,43]. The total sample size consisted of 1418 patients in the NS group and 1367 patients in the conventional CT-guided method group. Baseline characteristics are listed in Table 1. The PRISMA 2020 Flow Diagram is presented in Figure 1.

### 3.2. Number of Needle Manipulations

**Figure 2:**
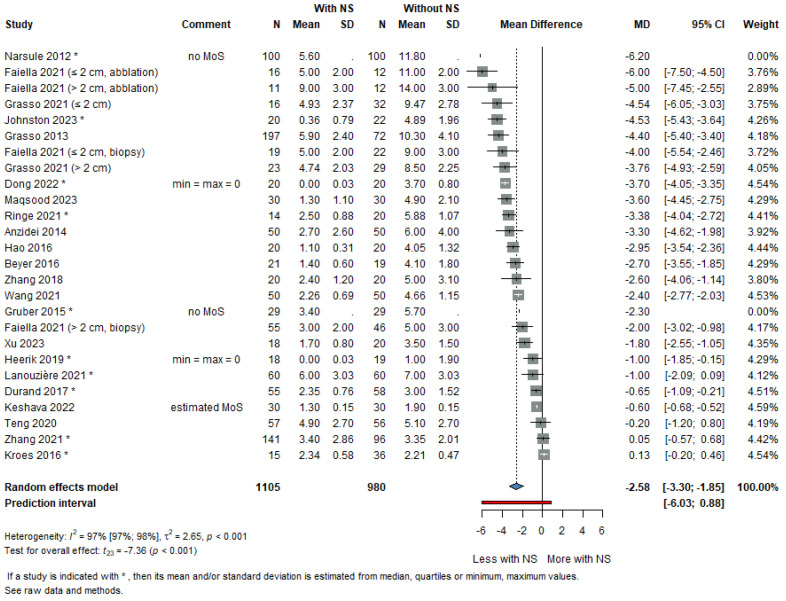
Pooled forest plot showing the mean difference (MD) in the number of needle manipulations [10,17,21,22,23,24,25,27,29,30,31,36,39].

The analysis included 24 studies covering a total of 2085 patients. Navigation systems significantly reduced the number of needle manipulations compared to conventional methods, with a pooled MD of −2.58 (95% CI: −3.30 to −1.85). The high heterogeneity (*I*^2^ = 97%) reflected variance in true effects rather than sampling error, and the prediction interval ranged from −6.03 to 0.88, suggesting that while benefit is likely, some future studies may observe minimal or no effect—Figure 2. This finding was consistent across subgroups. In the cohort subgroup (16 studies, 1213 patients), the MD was −3.24 (95% CI: −4.06 to −2.42; I^2^ = 91.8%), with a prediction interval of −6.44 to −0.05, indicating a robust benefit of navigation systems. In contrast, the RCT subgroup (7 studies, 821 patients) yielded a smaller but still significant reduction in needle manipulations (MD: −1.45, 95% CI: −2.53 to −0.37; I^2^ = 94.5%), with a prediction interval of −4.37 to 1.47—Supplemental Figure S1. Although the pooled estimate indicated a reduction in needle manipulations, the substantial between-study heterogeneity suggests that the magnitude of this effect varies across clinical settings and procedural contexts.

### 3.3. Procedural Time

The analysis included 28 studies covering 2659 patients. The pooled MD was −8.07 min (95% CI: −12.27 to −3.87), significantly reducing procedural time with navigation systems. The between-study heterogeneity (I^2^) was 99.1% (Supplemental Figure S2).

Supplemental Figure S3 shows that in the cohort subgroup, the MD was −10.25 min (95% CI: −15.94 to −4.56), with an I^2^ of 95%. The prediction interval ranged from −34.19 to 13.69. In the RCT subgroup, the MD was −4.37 min (95% CI: −10.85 to 2.11), with an I^2^ of 100%. The prediction interval ranged from −26.11 to 17.37. The test for differences between subgroups was not statistically significant (*p* = 0.135), indicating no significant difference in the effect of navigation systems on procedural time between RCTs and cohort studies.

**Figure 3:**
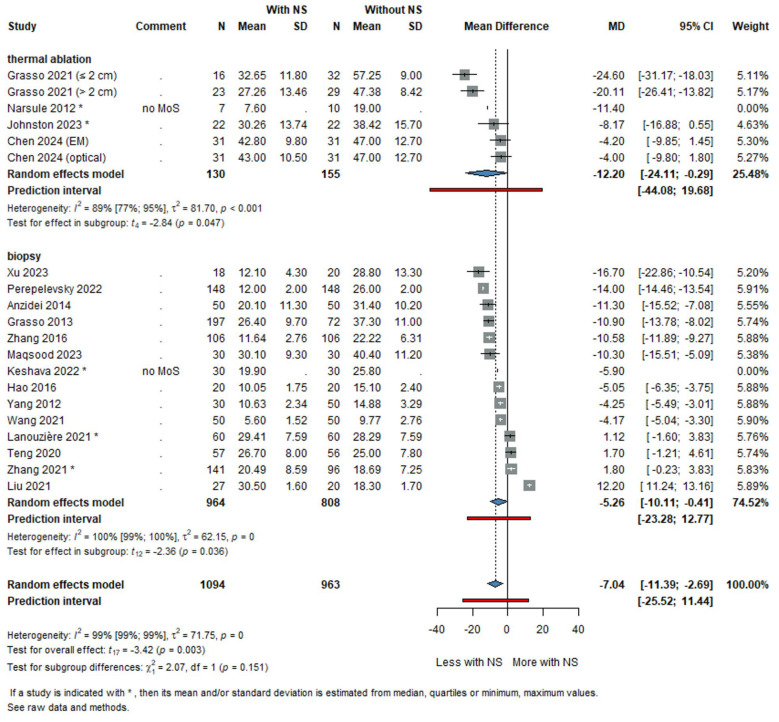
Forest plot showing the mean difference (MD) in thoracic procedural time between subgroups based on procedural type [9,15,16,19,20,21,24,25,27,30,31,32,33,35,37,38,39,40,42].

Subgroup analysis (Figure 3) based on procedural type revealed a significant reduction in procedural time for thoracic interventions, particularly for lung biopsy when using navigation systems, where the MD in procedural time was −5.26 min (95% CI: −10.11 to −0.41) in favour of NS. For thermal ablations, the reduction was also significant, with an MD of −12.20 min (95% CI: −24.11 to −0.29). Additionally, abdominal interventions (Supplemental Figure S4) showed a non-significant mean reduction of 0.23 min (95% CI: −9.71 to 10.16), indicating no clear benefit in this subgroup regarding procedural time. The high heterogeneity observed in this analysis indicates that the influence of navigation systems on procedural duration is likely modified by clinical and methodological factors, including target organ, intervention type, and operator experience.

### 3.4. Irradiation Dose

Twenty-four studies were included in the analysis of irradiation dose for a total of 1744 patients. The ROM in irradiation dose between groups using NS and those not using them was 0.63 (95% CI: 0.58 to 0.69). This indicates a statistically significant reduction in irradiation dose in the NS group, with a *p*-value of less than 0.001. The heterogeneity between studies was substantial, with I^2^ at 91% (95% CI: 89–94%).

For the cohort subgroup (14 studies, 1101 patients), the ROM was 0.64 (95% CI: 0.57 to 0.71), with I^2^ at 83% (95% CI: 73–88%). In the RCT subgroup (5 studies, 643 patients), the ROM was 0.62 (95% CI: 0.48 to 0.79), with I^2^ at 95% (95% CI: 91–97%).

The study by Heerink was identified as an outlier and excluded from the analysis [28]. This exclusion was due to the fact that the number of CT scans was the same in both groups, with the dose-length product (DLP) of the robotic group significantly higher, as a greater scan length was required to visualize all fiducials.

**Figure 4:**
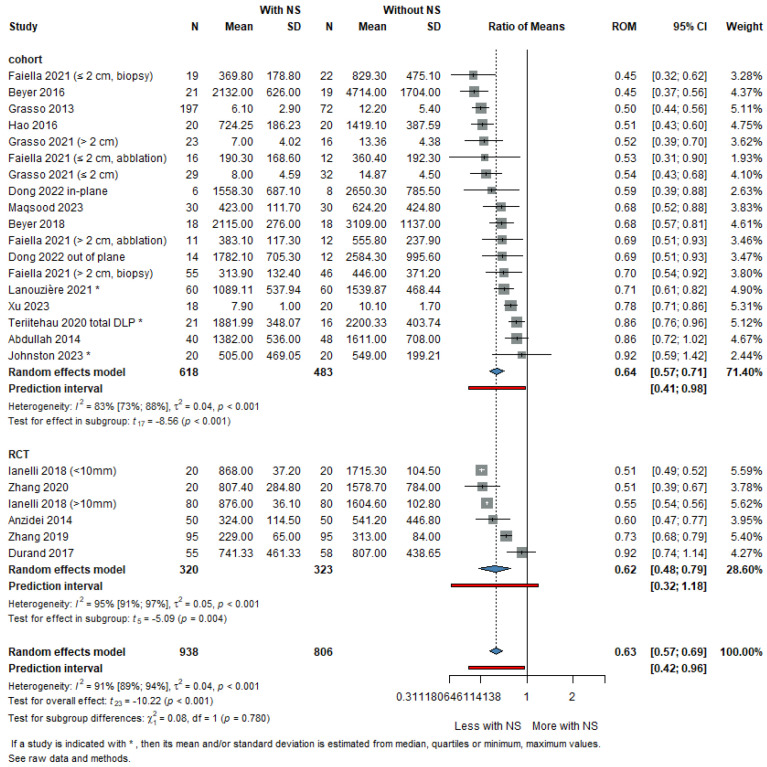
Forest plot showing the ratio of means (ROM) for irradiation dose between subgroups based on study type [9,10,15,16,17,21,22,24,25,27,29,30,33,38,39,41].

There were no statistically significant subgroup differences, with a *p*-value of 0.78 for the test of subgroup differences (Figure 4). While a reduction in radiation exposure was observed, the variability between studies implies that the extent of this benefit is not uniform and may depend on differences in navigation technology and imaging workflow.

Radiation exposure was most reported as DLP (mGy·cm), while a minority of studies used effective dose (mSv). Because of heterogeneity in reporting units, ROM was applied to enable pooled comparison across studies.

### 3.5. Complication Rates

**Figure 5:**
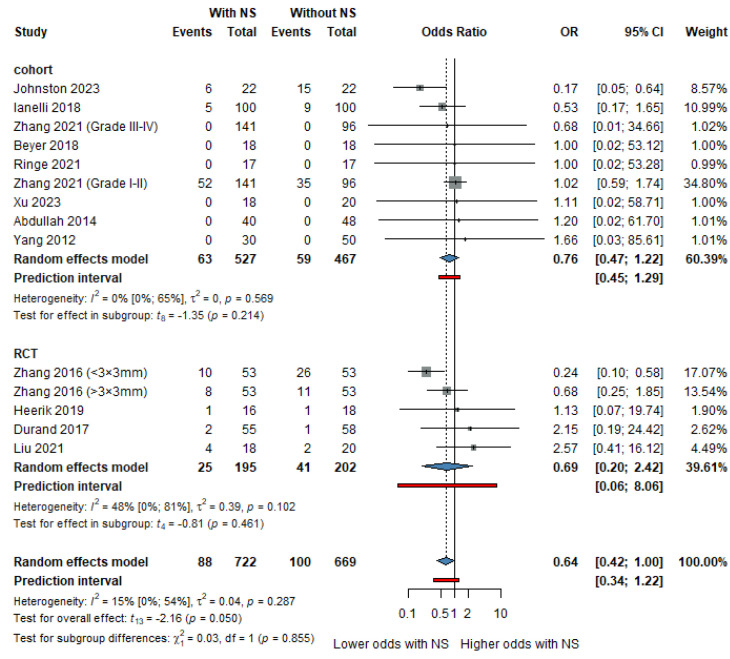
Forest plot showing the odds ratio (OR) of overall complication rates [15,17,22,28,29,30,32,36,37,39,40,42].

**Figure 6:**
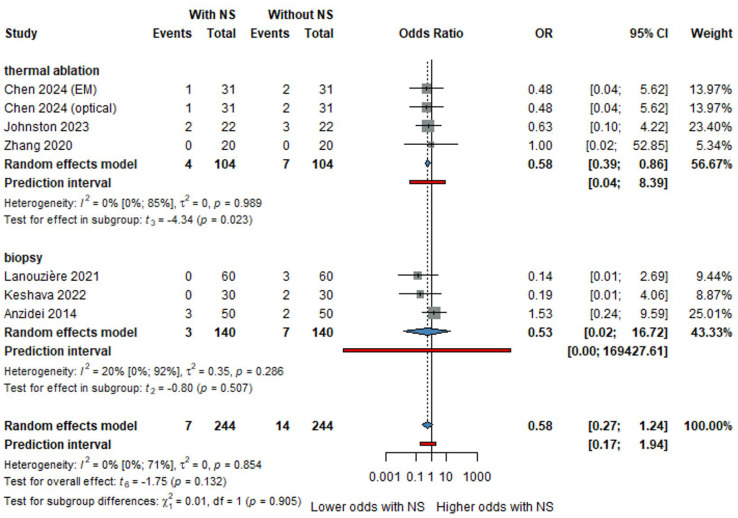
Forest plot showing the odds ratio (OR) for pneumothorax rates in lung thermal ablation and biopsy [9,16,19,30,31,38].

Navigation systems significantly reduced complication rates compared to conventional methods. The OR for overall complications was 0.64, as shown in Figure 5, with a non-significant reduction in the incidence of pneumothorax (Supplemental Figure S5, OR: 0.92, 95% CI: 0.61 to 1.38). In the thermal ablation subgroup, as shown in Figure 6, the OR for chest tube insertion was 0.58 (95% CI: 0.39 to 0.86, *p* = 0.023), further highlighting the safety benefits of navigation systems in reducing adverse events associated with needle insertions.

### 3.6. Success Rates

The analysis of the technical success rate (Supplemental Figure S6), which refers to the correct positioning of the probe or needle as confirmed by control CT scans, included 8 RCTs and 13 cohort studies, covering 1490 patients. The pooled OR for technical success was 1.41 (95% CI: 0.89 to 2.24), indicating a non-significant difference between groups. Between-study heterogeneity (I^2^) was 0%, with no variance of true effects (*τ*^2^ = 0). The prediction interval was 0.73 to 2.75. In the cohort subgroup, which included 13 studies with 988 patients, the OR was 1.3 (95% CI: 0.84 to 1.99), with an I^2^ of 0%. In the RCT subgroup, which included 7 studies with 502 patients, the OR was 1.47 (95% CI: 0.45 to 4.80), with an I^2^ of 9%. The test for subgroup differences was not statistically significant (*p* = 0.805).

The analysis of the diagnostic success rate (Supplemental Figure S7), defined as obtaining a positive histopathological result in biopsies, included 4 RCTs and 6 cohort studies covering 1342 patients. The pooled OR for diagnostic success was 1.66 (95% CI: 1.01 to 2.73), indicating a statistically significant difference in favor of navigation systems. Between-study heterogeneity (I^2^) was 0%, with no variance in true effects (*τ*^2^ = 0). The prediction interval ranged from 0.93 to 2.97. In the cohort subgroup, which included 6 studies with 799 patients, the OR was 1.48 (95% CI: 1.15 to 1.90), with an I^2^ of 0%. In the RCT subgroup, which included 4 studies with 543 patients, the OR was 2.25 (95% CI: 0.27 to 18.92), with an I^2^ of 49%. The test for subgroup differences was not statistically significant (*p* = 0.536). Across studies, absolute diagnostic success rates generally ranged between approximately 85% and 95%, supporting the clinical relevance of the observed effect.

The study by Zhang et al. was excluded from this analysis because their reported technical success rate only accounted for first-attempt success, while subsequent attempts were not distinguished [40]. This created a discrepancy where patients who were eventually successfully targeted were classified differently in technical versus diagnostic success categories, rendering the overall technical success rate unclear.

### 3.7. Risk of Bias Assessment and GRADE Assessment

The risk of bias was assessed using the revised Cochrane risk-of-bias tool for randomized trials (RoB 2) and the Risk Of Bias In Non-randomized Studies—of Interventions (ROBINS-I). The assessment showed a moderate to high risk of bias across several studies. Common issues included inadequate randomization leading to selection bias, differential outcome measurement causing detection bias, and selective outcome reporting contributing to reporting bias. Detailed visual summaries of the risk of bias assessment are provided in Supplemental Figure S8.

Overall, the level of evidence was rated as moderate due to the presence of these biases. The GRADE assessment tables for each outcome parameter are provided in Supplemental Table S3.

We conducted a leave-one-out sensitivity analysis that identified the Heerink study as an outlier in the analysis of irradiation dose [28]. A visual inspection of the funnel plots (Supplemental Figure S8) revealed no evidence of publication bias.

## 4. Discussion

Our meta-analysis included 30 studies (RCTs and cohort studies) with 2785 patients, comparing navigation systems to conventional freehand CT-guided methods on outcomes such as needle manipulations, radiation dose, procedural time, complication rates, and success rates. CT fluoroscopy-guided interventions were excluded due to methodological differences, as fluoroscopy involves continuous real-time imaging, resulting in higher radiation exposure than the stepwise freehand approach. Despite the consistent direction of effect across outcomes, the substantial heterogeneity observed in several analyses indicates that the magnitude of benefit is context dependent. Differences in target organ, navigation system type, operator experience, and study design likely contributed to this variability, and therefore pooled quantitative estimates should be interpreted with appropriate caution.

Despite statistical significance for several pooled outcomes, substantial clinical and methodological heterogeneity constrains the precision of the quantitative estimates. Variation in target organ, navigation system technology, procedural workflow, and operator experience implies that effect sizes may differ meaningfully across settings and patient populations. Accordingly, these findings should primarily be interpreted as supporting the direction of effect, rather than a single universally applicable magnitude of benefit. Subgroup analyses by study design demonstrated consistent direction of effect across randomized and observational studies, suggesting that the observed findings were not solely driven by studies with higher risk of bias.

This study extends existing literature by evaluating navigation systems as workflow-modifying tools rather than isolated technical solutions. By examining multiple procedural outcomes together, it highlights how improvements in targeting accuracy may translate into downstream effects on radiation exposure, efficiency, and complication risk across different clinical settings.

### 4.1. Number of Needle Manipulations:

A reduction in needle manipulations with navigation systems is consistent with improved targeting accuracy and fewer iterative trajectory adjustments. Clinically, fewer repositioning steps may translate into less tissue trauma and fewer control scans, which can influence both procedural efficiency and complication risk [44,45,46].

Significantly, manipulation with more needles is associated with prolonged procedural times, as each adjustment requires a control CT scan, increasing the overall duration and radiation exposure. This affects procedural efficiency and may influence the success rate, as prolonged procedures and repeated adjustments may compromise the accuracy of tissue sampling or ablation, ultimately affecting clinical outcomes. Navigation systems may contribute to shorter procedures, lower radiation exposure, and improved procedural performance by reducing the need for repeated needle manipulations.

Consistency across study designs supports the interpretation that navigation systems are associated with fewer needle adjustments. The reduction in the need for needle adjustments reflects the improved accuracy of navigation systems, which allow more precise targeting of lesions even in challenging anatomical locations. However, the magnitude of this reduction is likely to vary with lesion characteristics, respiratory motion (in thoracic procedures), and differences in navigation technology and operator experience.

### 4.2. Procedural Time

The observed reductions in procedural time suggest improved procedural efficiency; however, clinical relevance is context dependent. Time savings are expected to be more meaningful in shorter procedures (e.g., lung biopsy) than in longer, more complex interventions (e.g., thermal ablation), where setup requirements and workflow integration may offset gains. NS can shorten active intervention time by increasing precision and reducing the need for repeated adjustments. For instance, Gupta emphasized that NS minimizes the need for multiple needle manipulations and repeat scans by allowing more accurate lesion targeting [47].

However, the initial setup of NS can sometimes prolong the total procedural time, as Gupta noted. This trade-off between preparation and execution must be managed effectively to maximize the benefits of NS [48]. Similarly, CIRSE guidelines emphasize that integrating navigation systems into workflows requires careful planning to avoid inefficiencies associated with setup [2,3].

The operator’s experience also has a significant impact on procedural time. Theilig reported that procedural times decrease as operators become more proficient with NS, suggesting that training and knowledge are crucial for achieving efficiency gains [48].

The results of our analysis suggest that navigation systems are associated with reduced procedural time, consistent with prior evidence highlighting their efficiency benefits. This reduction may translate into shorter intervention time for patients, potentially improving comfort and minimizing the duration of radiation exposure.

Subgroup analyses allowed us to determine where navigation systems benefit most and better understand their performance across clinical scenarios.

The subgroup analysis showed different effects depending on the type of thoracic procedure. For lung thermal ablation, the MD in procedural time between groups was approximately 12 min (Figure 2), highlighting a significant reduction in time when navigation systems were used. However, given that ablation procedures are typically long and complex, lasting more than an hour on average, this reduction, although statistically significant, may have a limited clinical impact. The time savings, although notable, may not drastically change the overall workflow in the context of thermal ablation.

In lung biopsies, navigation systems have a more pronounced clinical impact. Lung biopsies, particularly those involving multiple needle adjustments, can take anywhere between 30 to 60 min [49], depending on whether a lesion is available and whether accurate positioning is required for each adjustment [50].

The approximately 5 min (Figure 2) reduction observed using navigation systems is clinically significant, representing a 10–15% decrease in total procedural time. Thus, navigation systems may improve biopsy workflows by minimizing both the number of needle manipulations and the overall duration of the procedure.

In abdominal interventions, the impact of navigation systems on procedural time may be less pronounced, as many procedures are already performed under ultrasound guidance and are typically shorter in duration.

Compared to RCTs, the more substantial effect observed in cohort studies (Supplemental Figure S4) may be attributed to the larger patient population and clinical environments represented in observational designs. In cohort studies, there is more variability in factors such as lesion size, experience of the operator, and patient health status. These variations are likely to enhance the observed impact of navigation systems as their benefits become more apparent when addressing a wider range of procedural challenges. This leads to a more significant reduction in procedural time when navigation systems are introduced into routine clinical practice. Crucially, the results of both subgroups align in favor of navigation systems. Consistency across study designs suggests that navigation systems may improve procedural efficiency in controlled environments and wider clinical practice.

Differences in workflow (patient positioning, respiratory coaching, control scan strategy), the learning curve, and the time required for device setup/calibration likely explain why procedural-time effects are more variable than effects on needle manipulations.

### 4.3. Irradiation Dose

Although 85% of the total radiation dose is usually delivered during the pre-and post-intervention CT scans, and only 15% is attributed to the CT-guided procedure itself, navigation systems were associated with reduced radiation exposure by decreasing the need for repeated needle adjustments and subsequent control scans [51]. This reduction contributes to a significant decrease in cumulative radiation dose, with a ROM of 0.63 (95% CI: 0.58 to 0.69), as shown in Figure 3, corresponding to a relative reduction in radiation dose of nearly 40%.

Although navigation systems are effective in reducing overall radiation dose, their use may require adjustments that may offset some of these benefits. For instance, Heerink [28] observed that while the number of CT scans was similar between robotic and freehand procedures, the DLP was significantly higher in the robotic group due to the greater scan length required to accommodate fiducials. This highlights the trade-off between the precision offered by NS and the potential increase in irradiation dose, underscoring the need for optimized imaging protocols to maximize the benefits of NS and minimize radiation exposure.

### 4.4. Complication Rates

Abdominal interventions, for example, may involve retroperitoneal bleeding, infection, or injury to adjacent organs. At the same time, musculoskeletal procedures are more likely to result in minor issues such as paresthesia, bone fissures, or minor bleeding [46].

Thoracic interventions, particularly lung biopsies and thermal ablation, are associated with a higher incidence of pneumothorax (PTX) and hemorrhage [52]. Najafi evaluated the PEARL approach for CT-guided lung biopsy and highlighted optimized methods to minimize complication rates, underlining the need for procedural precision to reduce risks [53].

Heerink conducted a meta-analysis on CT-guided lung biopsies, providing comprehensive insights into complication rates and reinforcing the importance of adopting strategies to mitigate PTX and other risks during lung interventions collectively underscore the necessity of careful planning, real-time monitoring, and the use of advanced techniques like navigation systems to minimize complication rates in CT-guided procedures [28].

Our meta-analysis shows that navigation systems showed a trend toward lower overall complication rates, indicating a trend toward reduced complications, although not reaching statistical significance. Notably, cohort studies and RCTs align in their findings, suggesting that navigation systems can contribute to reducing complications, as shown in Figure 4.

To further understand the impact of navigation systems, we assessed PTX rates, a common complication in lung interventions such as biopsies. The analysis revealed no significant difference in PTX rates between the study and control groups (Figure 5). This likely reflects that pneumothorax risk is mainly determined by pleural traversal and lesion location, factors not fully modified by navigation. The reduced chest tube rate in thermal ablation suggests that navigation may influence pneumothorax severity rather than overall incidence. However, a notable finding emerged when examining the subgroups for thermal ablation and biopsy procedures: the need for chest tube insertion was significantly lower in the lung thermal ablation subgroup when NS was utilized. In the thermal ablation subgroup (208 patients, 4 studies), navigation systems significantly reduced the odds of chest tube insertion (OR: 0.58, 95% CI: 0.39 to 0.86, *p* = 0.023), as shown in Supplemental Figure S6. This highlights that while NS may not significantly reduce overall PTX rates, it can be critical in lowering more severe complications like chest tube insertion, particularly in thermal ablation settings, which may be clinically relevant for patient outcomes.

### 4.5. Success Rates

Our analysis of technical and diagnostic success rates suggests a potential advantage of NS in improving outcomes for patients undergoing CT-guided interventions. The technical success rate is often defined as the accurate placement of the biopsy needle or ablation probe at the target site, as confirmed by a post-positioning CT scan. Diagnostic success rate refers to the procurement of sufficient tissue for pathological diagnosis in case of biopsies.

The studies reported a technical success rate of NS procedures, which was consistently high (Figure 6). For example, studies such as those by Beyer and Liu show improved technical accuracy in precise needle positioning, even for smaller or more difficult-to-access lesions, reducing the need for multiple needle manipulations and potential repositioning [18,32]. The technical success rates of NS-assisted procedures range from 95% to 99% in several clinical series [52], demonstrating the high accuracy facilitated by real-time navigation systems. Studies such as Zhang (2021) and Perepelevsky (2022) show that NS improves diagnostic yield, especially for small or difficult-to-reach lesions [42]. The literature reports diagnostic success rates as high as 92% to 97% for CT-guided biopsies [52,53,54,55], which exceed conventional CT-guided techniques that sometimes struggle with smaller lesions or nodules deep in the lung. This is particularly relevant in biopsies, where obtaining an adequate tissue sample is crucial for a definitive diagnosis. The improved real-time visualization and accuracy of NS may contribute to higher diagnostic yield, reducing the need for repeat procedures and ensuring faster patient diagnosis.

Lung biopsies using the conventional method are challenging due to lesion movement during local anesthesia, requiring patient coordination with controlled respiration. This patient-dependent factor makes it difficult to achieve consistent outcomes. Although the study by Zhang was excluded due to unclear technical success rates, it highlighted the value of NS, reporting higher first-attempt success rates (69.8% vs. 22.7%, *p* < 0.01) and diagnostic accuracy (96.2% vs. 81.1%, *p* < 0.05) for small lesions, as well as fewer complications and shorter procedural times [40].

As emphasized in the study by Kim, fluoroscopy can mitigate lesion movement by providing real-time feedback, reducing procedural time and needle passes, and achieving diagnostic accuracy comparable to conventional CT-guided techniques, though at the cost of increased radiation exposure [5]. These findings suggest a potential role for navigation systems in managing small or difficult-to-access lesions.

### 4.6. Implications for Future Research Practice

Future research should prioritize operator skills and the comparative performance of navigation systems. Studies on how operator experience influences outcomes can provide guidance for standardized training. In addition, comparisons of NS technologies, such as electromagnetic, optical, and stereotactic systems, are needed to identify the best options for specific interventions, such as biopsies or ablations.

### 4.7. Strength and Limitations

The strength of this meta-analysis is that it includes diverse procedures, such as biopsies and ablations, across various target organs, such as the lung and liver, improving generalizability. Subgroup analyses provide valuable insights into NS performance, while the large patient population strengthens statistical power and reliability.

However, limitations include grouping diverse NS technologies, high heterogeneity in some analyses, and inconsistent consideration of the learning curve and operator experience. Variations in local procedural techniques and the absence of long-term outcome and cost-effectiveness data further limit the scope of the study for broader clinical and economic guidance.

Lesion size and lesion complexity are clinically relevant determinants of procedural difficulty; however, lesion size was inconsistently reported across the included studies (different metrics and summary formats), which precluded quantitative synthesis and limited subgroup exploration based on lesion size.

In addition, the absence of robust health economic evaluations represents an important limitation. Although navigation systems may offer procedural advantages, acquisition costs and workflow integration remain key determinants of real-world adoption. The lack of formal cost-effectiveness or budget impact analyses within the current evidence base limits conclusions regarding implementation and should be prioritized in future research.

This study was conducted within the translational medicine framework, as the Academia Europaea proposed. As such, it aims to bridge the gap between clinical science and medical practice [56,57].

## 5. Conclusions

This meta-analysis demonstrates that navigation systems significantly improve procedural efficiency and safety in CT-guided interventions. They reduce needle manipulations, procedural time, complication rates, and radiation exposure while increasing technical and diagnostic success rates. These findings support the wider adoption of navigation systems in clinical practice, recognizing their potential to improve patient care during a variety of interventions.

## Figures and Tables

**Table 1:** Basic characteristics of the included studies.

Study Author	Total N	NS/FH	Mean Age ± SD	Female (%)	Target Organ	Intervention Type	NS Type
Abdullah B.J.J. et al.	50	20/30	59.25 ± 11.9 (NS group) *	30% (NS group)	Hepatic tumors (HCC & metastases)	Thermal ablation	MAXIO Robotic System
Anzidei M. et al.	100	50/50	65 ± 4	37%	Pulmonary lesions	Biopsy	ROBIO™ EX Robotic Positioning System
Beyer L.P. et al.	36	18/18	63.3 ± 11.9 *	36%	Hepatic tumors (HCC & metastases)	Thermal ablation	CAS-One IR Stereotactic Navigation System
Beyer L.P. et al.	40	21/19	60.3 (range 46–78) *	17%	Hepatic tumors (HCC & metastases)	Thermal ablation	Perfint MAXIO Robotic System
Chen Z. et al.	70	50/20	Median 60 (range 16–85)	34%	Pulmonary lesions	Thermal ablation	Veran Electromagnetic Navigation System, XinAo MDT Optical Navigation System
Dong Y. et al.	40	20/20	55.2 ± 10.0	27%	Single HCC <3 cm	Thermal ablation	RapidNav Robotic Laser Positioning System (RLPS)
Durand P. et al.	120	60/60	60.6 ± 15.0 *	41%	Various CT-guided interventions (biopsy, drainage, ablation, etc.)	Biopsy, drainage, tumor ablation, sympathicolysis	IMACTIS Electromagnetic Navigation System
Faiella E. et al.	193	101/92	56.0 ± 14.6 *	50%	Bone lesions (primary and metastatic)	Biopsy, thermal ablation	SIRIO Augmented Reality Navigation System
Grasso R.F. et al.	101	52/49	67.9 ± 11.1 *	43%	Pulmonary lesions	Thermal ablation	SIRIO Augmented Reality Navigation System
Grasso R.F. et al.	269	197/72	67.4 ± 11.5 *	37%	Pulmonary lesions	Biopsy	SIRIO Augmented Reality Navigation System
Gruber-Rouh T. et al.	58	29/29	62.9 (range 39–87)	33%	Various CT-guided interventions (biopsy, drainage, etc.)	Biopsy, drainage	Laser Navigation System
Hao W. et al.	40	20/20	66 (range 46–82)	35%	Pulmonary lesions	Biopsy	IG4 Electromagnetic Navigation System
Heerink W.J. et al.	39	18/19	60 (range 25–88) *	55%	Hepatic tumors (HCC & metastases)	Thermal ablation	Needle Positioning System (DEMCON Advanced Mechatronics)
Iannelli G. et al.	200	100/100	70.5 (spread not reported)	29%	Pulmonary lesions	Biopsy	SIRIO Augmented Reality Navigation System
Johnston E.W. et al.	39	20/20	65 ± 13	48%	Pulmonary lesions	Thermal ablation	Robotic—Perfint MAXIO, Perfint Pvt, Chen- nai, IN)
Keshava S.N. et al.	60	30/30	50.5 (spread not reported)	NA	Pulmonary lesions	Biopsy	HigHNoon Shadow-Based Needle Positioning System
Lanouzière M. et al.	120	60/60	67 (range 58–73) *	49%	Pulmonary lesions	Biopsy	IMACTIS Electromagnetic Navigation System
Liu Q. et al.	47	27/20	median 56 (IQR:48–61) *	45%	Pulmonary lesions	Biopsy	Savior-L Electromagnetic Navigation System
Maqsood S. et al.	60	30/30	63 ± 4	20%	Pulmonary lesions	Biopsy	MAXIO Robotic System
Narsule C.K. et al.	17	7/10	72 (range 60–84)	41%	Pulmonary lesions	Biopsy, thermal ablation	Veran Electromagnetic Navigation System
Perepelevskiy A.N. et al.	296	148/148	64.1 ±9.6	36%	Pulmonary lesions	Biopsy	Coaxial System with Permanent Infiltration Anesthesia
Ringe K.I. et al.	34	17/17	63 ± 12	48%	Hepatic tumors (HCC & metastases)	Thermal ablation	IMACTIS Electromagnetic Navigation System
Teng J. et al.	113	57/56	61.8 ± 9.3 *	38%	Pulmonary lesions	Biopsy	LungCare Electromagnetic Navigation System
Wang Y. et al.	100	50/50	55.1 ± 12.3	48%	Pulmonary lesions	Biopsy	Remote Laser-Guided Needle Angle Device
Xu H. et al.	38	18/20	68.9 (range 50–85)	42%	Pulmonary lesions	Biopsy	Laser Angle Selection System (LASS)
Yang J. et al.	80	30/50	57.9 ± 8.7 *	31%	Pulmonary lesions	Biopsy	IG4 Electromagnetic Navigation System
Zhang K. et al.	212	106/106	53.5 * (range 17–82)	40%	Pulmonary lesions	Biopsy	MSCT Three-Dimensional Digital Navigated Puncture Technique
Zhang L. et al.	200	100/100	53 ± 13	74%	Pulmonary lesions	Lung nodule localization	3D-Printed Navigational Template
Zhang Y. et al.	237	141/96	median 65 (range 32–80)	43%	Pulmonary lesions	Biopsy	LungCare Electromagnetic Navigation System
Zhang Z. et al.	40	20/20	57 (range 34–71)	10%	Liver tumors (≤5 cm)	Thermal ablation	IG4 Electromagnetic Navigation System

NS = Navigation system; FH = freehand; HCC = Hepatocellular carcinoma; RFA = Radiofrequency ablation; MWA = Microwave ablation; IQR = Interquartile range; SD = Standard deviation; EM = Electromagnetic; CT = Computed tomography; MSCT = Multislice computed tomography; 3D = Three-dimensional. Note: Age is reported as mean ± standard deviation unless otherwise specified. If reported as median (range) or median [IQR], the format is indicated. Values marked with an asterisk (*) are estimated by the authors based on subgroup data or calculations where exact values were not directly reported.

## Data Availability

All data supporting the findings of this study are available within the article and its Supplemental Digital Content.

## Supplementary Materials

The following supporting information can be downloaded at: https://www.mdpi.com/article/10.3390/life16030431/s1. Figure S1: Forest plot showing the mean difference in the number of needle manipulations by study design; Figure S2: Forest plot showing the pooled mean difference in procedural time; Figure S3: Forest plot of procedural time stratified by study design; Figure S4: Forest plot showing the mean difference in abdominal procedural time; Figure S5: Forest plot showing odds ratios for chest tube insertion in lung thermal ablation and biopsy subgroups; Figure S6: Forest plot showing the odds ratio for technical success rates; Figure S7: Forest plot showing the odds ratio (OR) for diagnostic success rates; Figure S8: Risk of bias summary; Table S1: Detailed search strategies across databases; Table S2: PRISMA 2020 checklist; Table S3: GRADE summary of findings.

## Abbreviation

The following abbreviations are used in this manuscript:

AR	Augmented reality
CI	Confidence interval
CT	Computed tomography
CTF	Computed tomography fluoroscopy
DLP	Dose-length product
EM	Electro magnetic
GRADE	Grading of Recommendations Assessment, Development and Evaluation
IR	Interventional radiology
MD	Mean difference
NS	Navigation system
OR	Odds ratio
PRISMA	Preferred Reporting Items for Systematic Reviews and Meta-Analyses
RCT	Randomized control trial
ROBINS-I	Risk of bias in non-randomized studies of interventions
RoB 2	Revised Cochrane risk-of-bias tool for randomized trials
ROM	Ratio of means
SD	Standard deviation
