# Supplementary material for: Navigation Systems Significantly Improve the Efficiency and Safety of CT-Guided Interventions
Source: Life (Basel). 2026 Mar 6;16(3):431. doi: 10.3390/life16030431 (PMC13027737; doi:10.3390/life16030431)
Supplement: Supplementary file 1 [file life-16-00431-s001.zip › Supplemental Figures.pdf]

Supplemental Figures

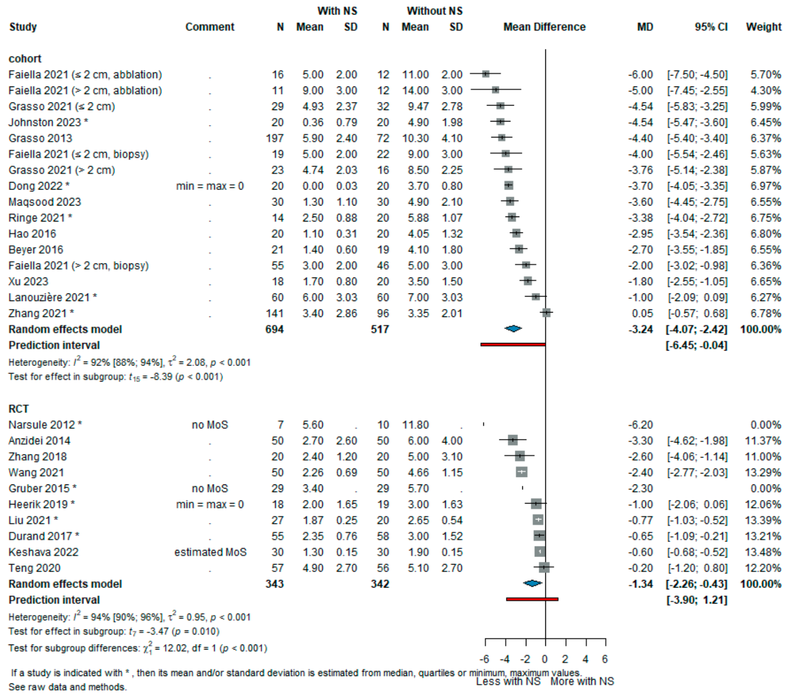

Supplemental Figure S1. Forest plot showing the mean difference in the number of needle manipulations by study design.

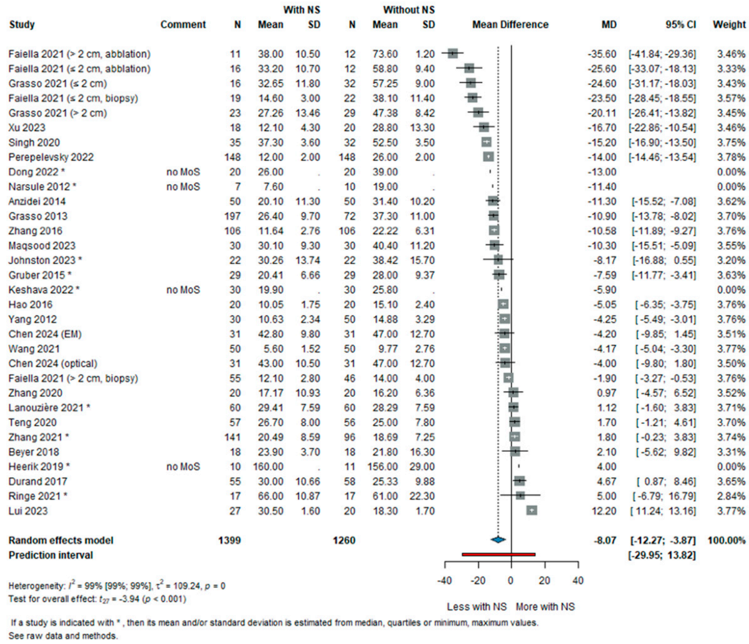

Supplemental Figure S2. Forest plot showing the pooled mean difference in procedural time.

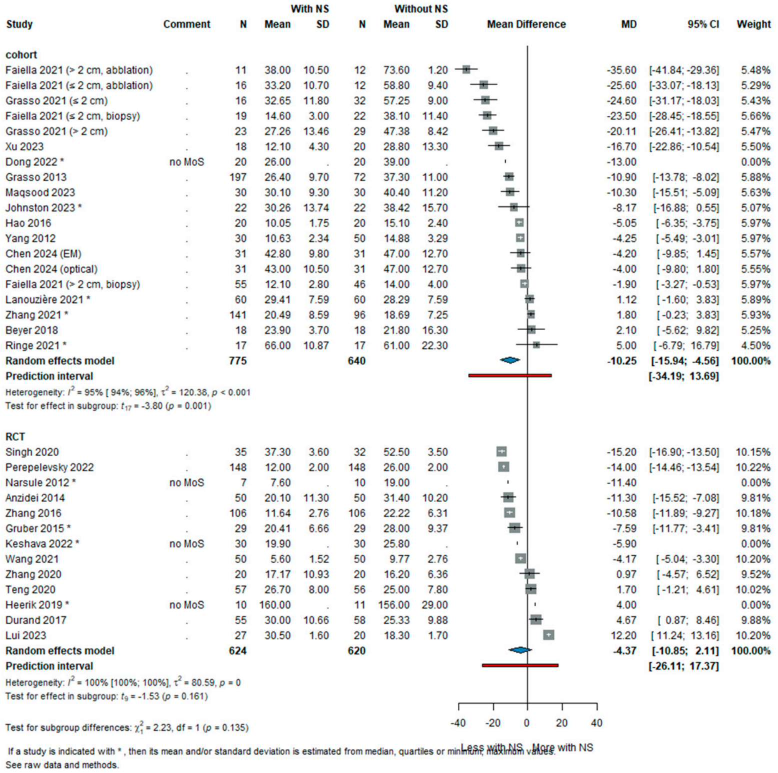

Supplemental Figure S3. Forest plot of procedural time stratified by study design.

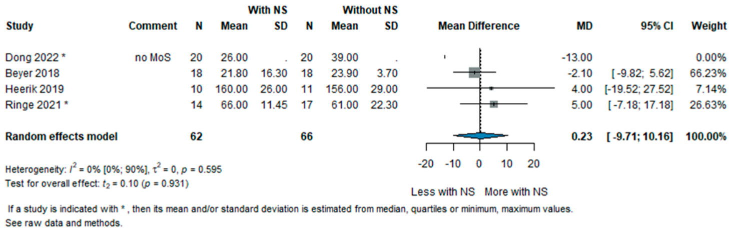

Supplemental Figure S4. Forest plot showing the mean difference in abdominal procedural time.

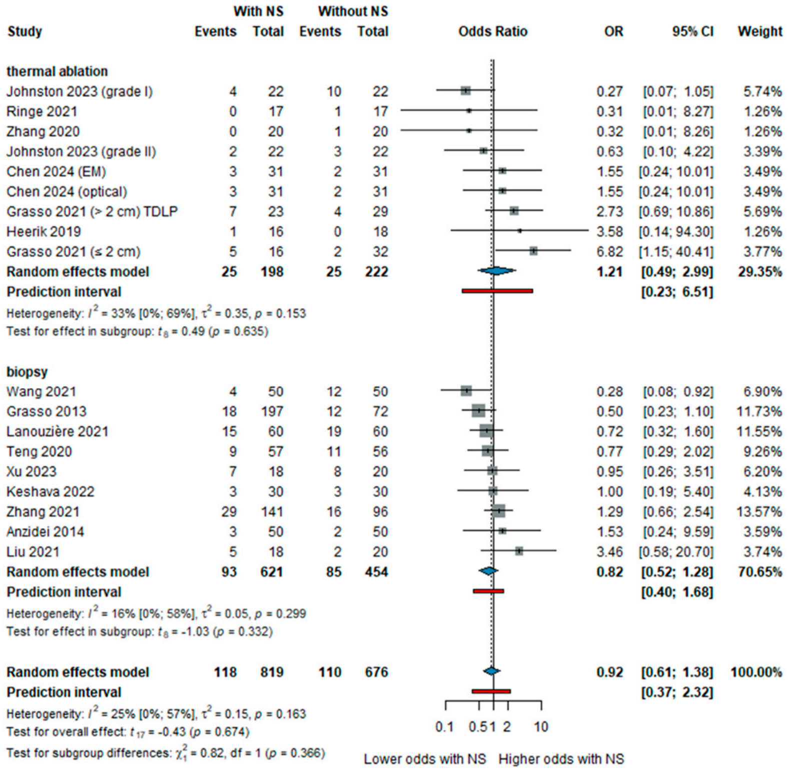

Supplemental Figure S5. Forest plot showing odds ratios for chest tube insertion in lung thermal ablation and biopsy subgroups.

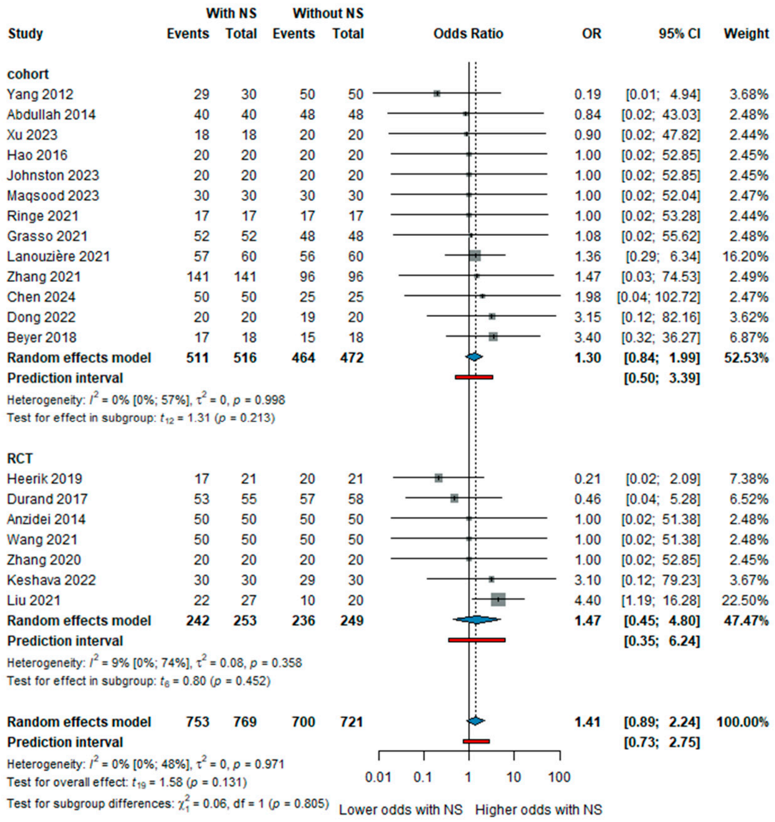

Supplemental Figure S6. Forest plot showing the odds ratio for technical success rates.

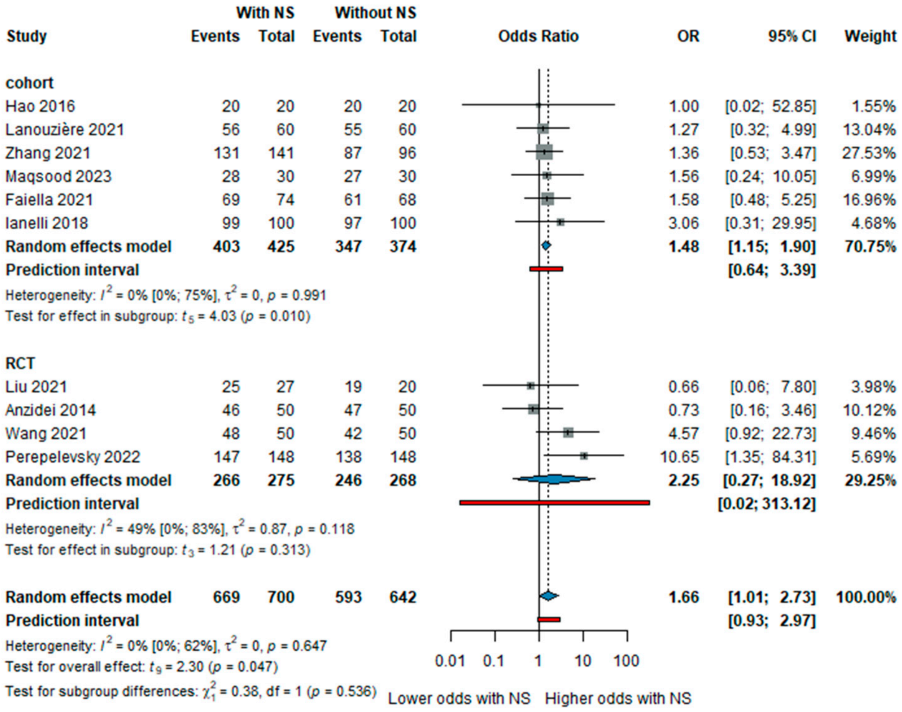

Supplemental Figure S7. Forest plot showing the odds ratio for diagnostic success rates.

Supplemental figure S7. Risk of Bias summary

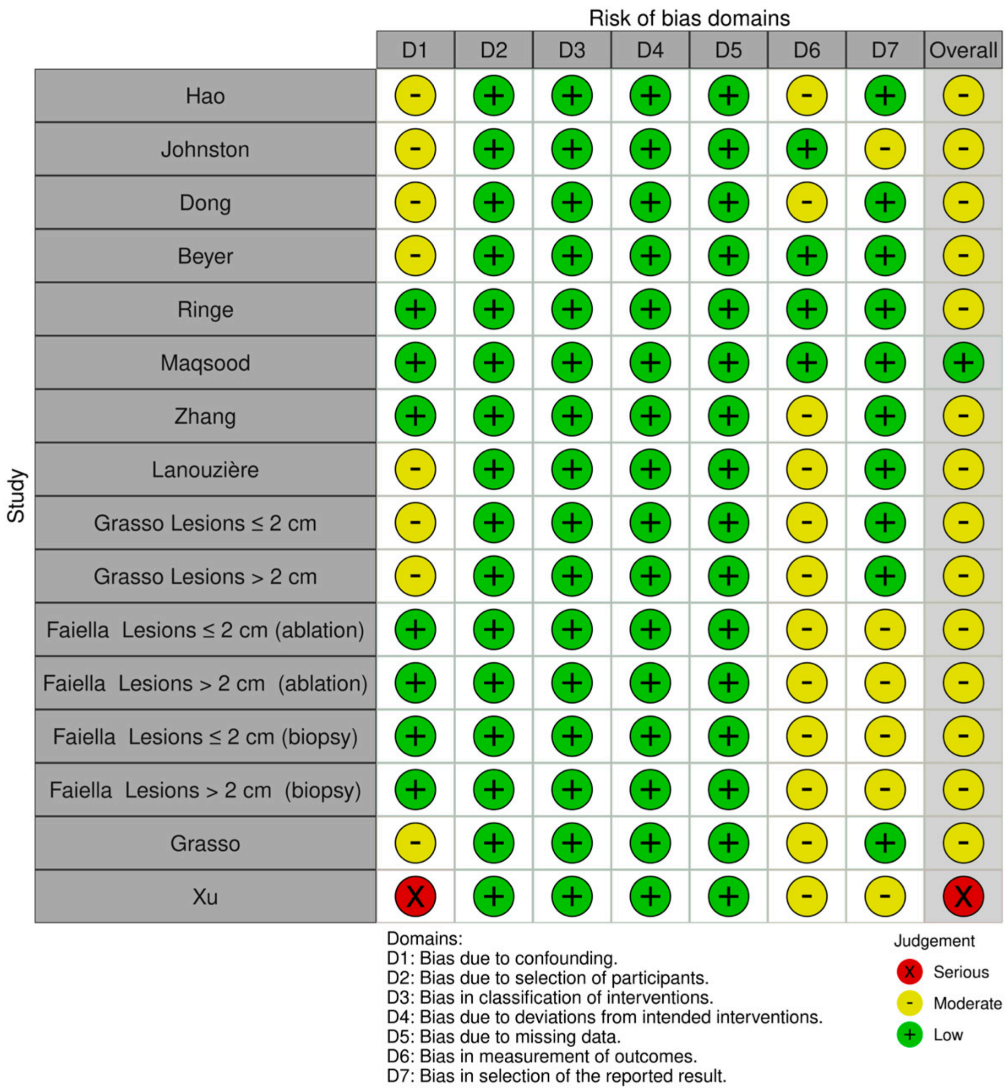

S7a. Risk of bias – Needle manipulations (ROBINS-I)

Study ID	D1	D2	D3	D4	D5	Overall
Narsule C.K. et al.	-	+	+	+	!	-
Anzidei M. et al.	!	+	+	+	!	!
Zhang Z. et al.2018	!	+	+	+	!	!
Wang Y. et al.2021	!	+	+	+	!	!
Gruber-Rouh T. et al.2015	!	+	+	+	!	!
Keshava S.N. et al.2022	!	+	+	+	!	!
Heerink W.J. et al.2018	+	+	+	+	!	!
Durand P. et al.2017	+	+	+	+	!	!
Teng J. et al.2020	+	+	+	+	!	!

S7b. Risk of bias – Needle manipulations (RoB 2)

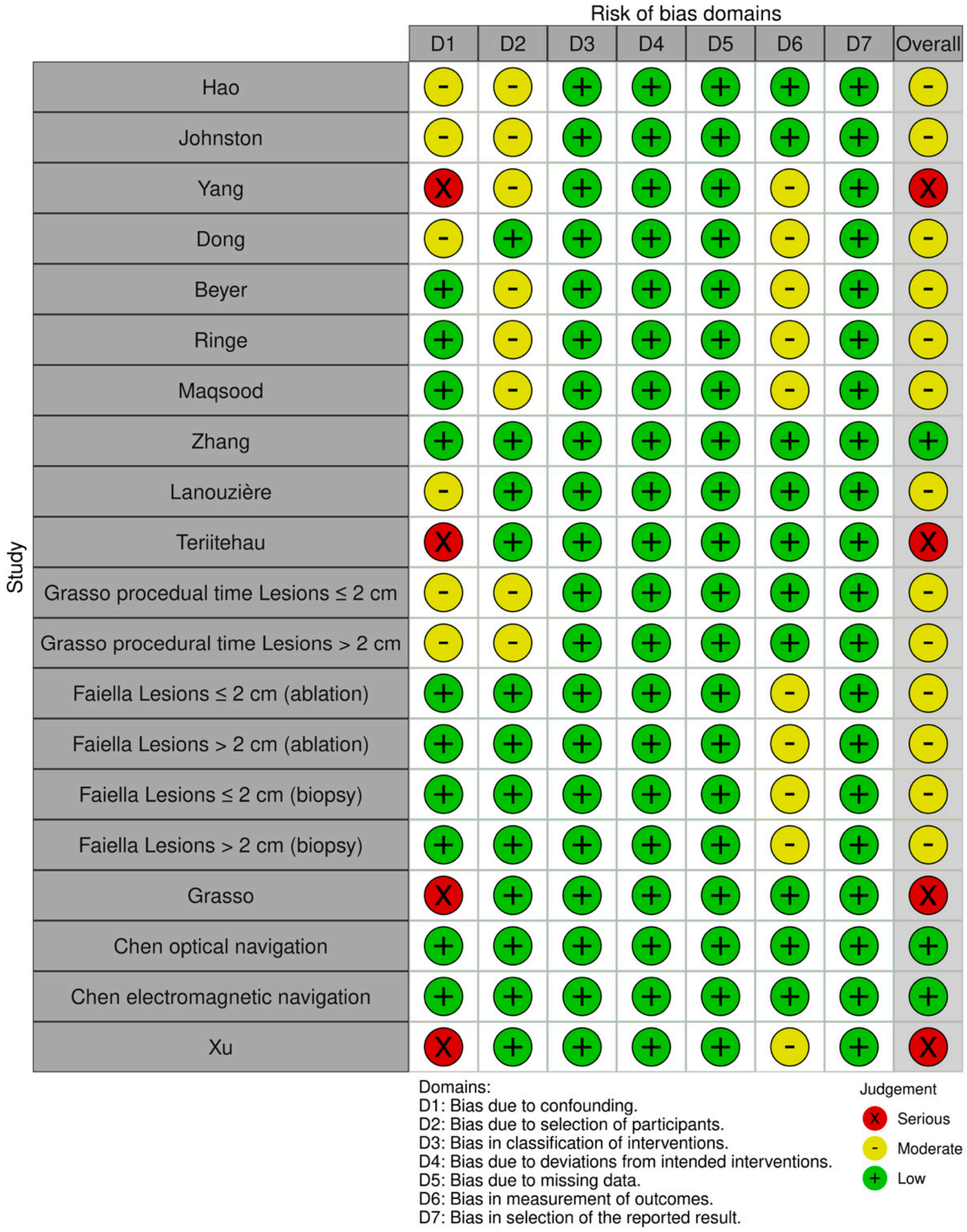

S7c. Risk of bias – Procedural time (ROBINS-I)

Study ID	D1	D2	D3	D4	D5	Overall
Teng J. et al.2020	+	+	+	+	!	!
Keshava S.N. et al.2022	+	+	+	+	!	!
Durand P. et al.2017	+	+	+	+	!	!
Heerink W.J. et al.2018	+	+	+	+	!	!
Gruber-Rouh T. et al.2015	!	+	+	+	!	!
Anzidei M. et al.2015	!	+	+	+	!	!
Zhang Z. et al.2018	+	+	+	+	!	!
Wang Y. et al.2021	!	+	+	+	!	!

S7d. Risk of bias – Procedural time (RoB 2)

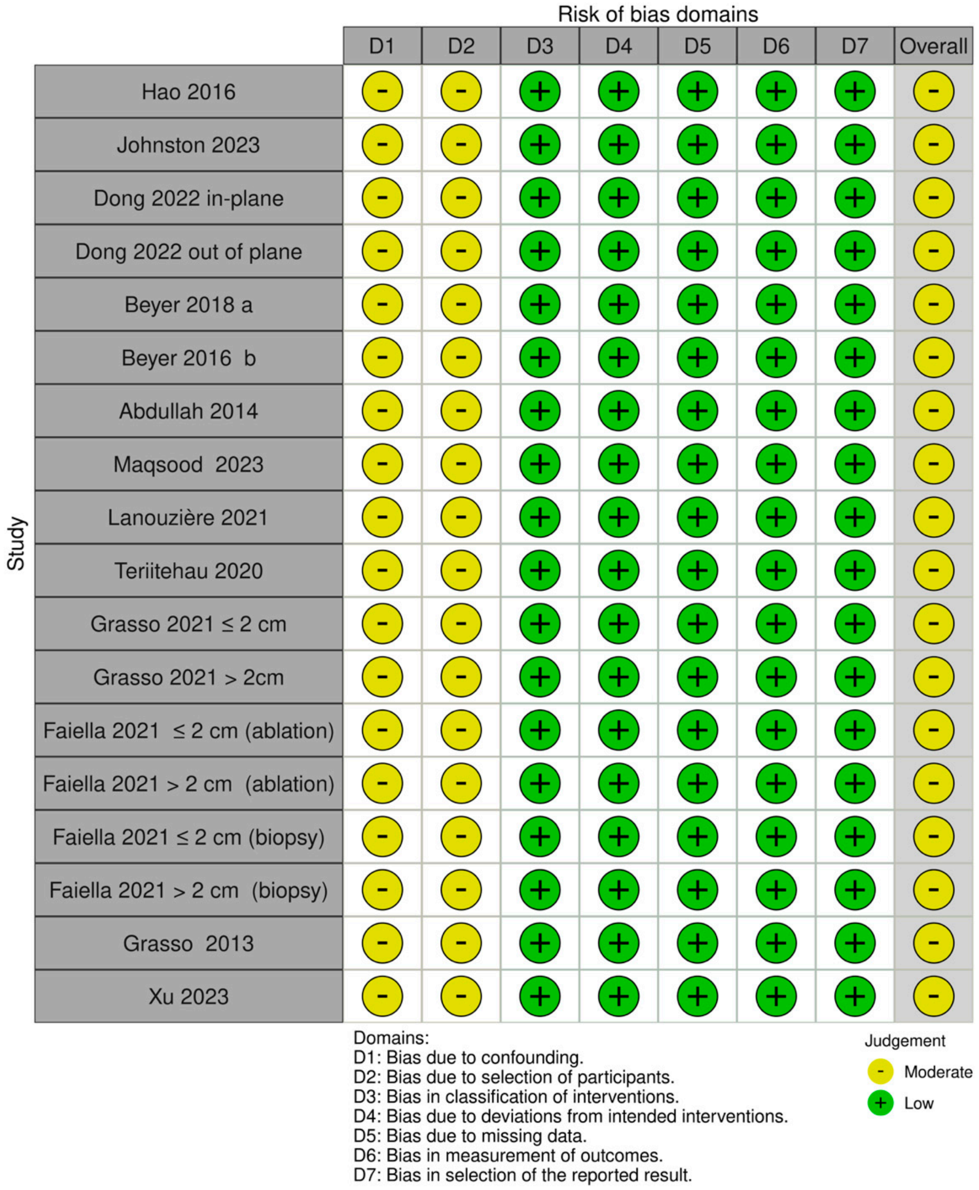

S7e. Risk of bias – Irradiation dose (ROBINS-I)

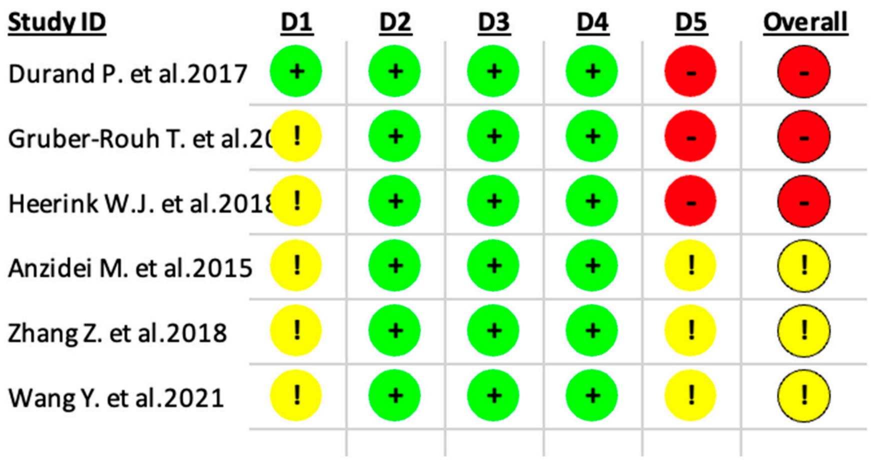

S7f. Risk of bias – Irradiation dose (RoB 2)

Risk of bias domains

Study	D1	D2	D3	D4	D5	D6	D7	Overal
Hao, major complication	-	-	+	+	+	+	?	-
Johnston, all	-	-	+	+	+	+	-	-
Johnston, pneumothorax	-	-	+	+	+	+	-	-
Johnston, chest tube insertion (with pneumothorax)	-	-	+	+	+	+	-	-
Johnston, dyspnea	-	-	+	+	+	+	-	-
Johnston, multiterritory embolic stroke	-	-	+	+	+	+	-	-
Yang, all	-	-	+	+	+	+	X	X
Zhang 2020, major complication	-	-	+	+	+	+	?	-
Zhang 2020, subcapsular haematoma	-	-	+	+	+	+	?	-
Zhang 2020, pneumothorax	-	-	+	+	+	+	?	-
Zhang 2020, chest tube insertion (with pneumothorax)	-	-	+	+	+	+	?	-
Dong, major complication	-	-	+	+	+	+	+	-
Dong, minor complication	-	-	+	+	+	+	+	-
Dong, diarrhea	-	-	+	+	+	+	+	-
Dong, fever	-	-	+	+	+	+	+	-
Dong, intrahepatic bleeding	-	-	+	+	+	+	+	-
Dong, abdominal pain	-	-	+	+	+	+	+	-
Beyer, all	-	-	+	+	+	-	+	-
Abdullah, all	+	-	+	+	+	+	+	+
Ringe, subcapsular haematoma	+	-	+	+	+	-	-	-
Ringe, hemorrhage Grade I.	+	-	+	+	+	-	-	-
Ringe, hemorrhage Grade II.	+	-	+	+	+	-	-	-
Ringe, pneumothorax	+	-	+	+	+	-	-	-
Ringe, all	+	-	+	+	+	-	-	-
Maqsood , major complication (and hemothorax)	+	-	+	+	+	-	+	-
Zhang 2021, all Grade I-II	+	-	+	+	+	-	+	-
Zhang 2021, pneumothorax	+	-	+	+	+	-	+	-
Zhang 2021, hemorrhage	+	-	+	+	+	-	+	-
Zhang 2021, hemoptysis	+	-	+	+	+	-	+	-
Zhang 2021, all Grade III-IV	+	-	+	+	+	-	+	-
Lanouzière , major complication	-	-	+	+	+	-	+	-
Lanouzière , pneumothorax	-	-	+	+	+	-	+	-
Lanouzière , chest tube insertion (with pneumothorax)	-	-	+	+	+	-	+	-
Lanouzière , thoracic wall hematoma	-	-	+	+	+	-	+	-
Lanouzière , hemothorax	-	-	+	+	+	-	+	-
Lanouzière , hemorrhage	-	-	+	+	+	-	+	-
Lanouzière , hemoptysis	-	-	+	+	+	-	+	-
Lanouzière , systemic air embolisation	-	-	+	+	+	-	+	-
Teriitehau , major complication	-	-	+	+	+	-	+	-
Grasso , pneumothorax,procedure time Lesions ≤ 2 cm	-	-	+	+	+	-	+	-
Grasso , pleural effusion,procedure time Lesions ≤ 2 cm	-	-	+	+	+	-	+	-
Grasso , hemopericardium,procedure time Lesions ≤ 2 cm	-	-	+	+	+	-	+	-
Grasso , broncho-pleural fistula,procedure time Lesions ≤2 cm	-	-	+	+	+	-	+	-
Grasso , pneumothorax,procedure time Lesions > 2 cm	-	-	+	+	+	-	+	-
Grasso , pleural effusion,procedure time Lesions > 2 cm	-	-	+	+	+	-	+	-
Grasso , massive subcutaneus emphysaema,procedure time Lesions > 2 cm	-	-	+	+	+	-	+	-
Grasso , broncho-pleural fistula,procedure time Lesions > 2 cm	-	-	+	+	+	-	+	-
Faiella , minor complication	-	-	+	+	+	-	+	-
Faiella , major complication	-	-	+	+	+	-	+	-
Grasso , pneumothorax	-	-	+	+	+	-	+	-
Grasso , hemorrhage	-	-	+	+	+	-	+	-
Grasso , hemoptysis	-	-	+	+	+	-	+	-
Grasso , hemothorax	-	-	+	+	+	-	+	-
a Chen, pain	-	-	+	+	+	-	+	-
a Chen, hemoptysis	-	-	+	+	+	-	+	-
a Chen, pneumothorax	-	-	+	+	+	-	+	-
a Chen, chest tube insertion (with pneumothorax)	-	-	+	+	+	-	+	-
Chen, fever, optical navigation	-	-	+	+	+	-	+	-
Chen, major complication, optical navigation	-	-	+	+	+	-	+	-
Chen, pain, optical navigation	-	-	+	+	+	-	+	-
Chen, hemoptysis, electromagnetic navigation	-	-	+	+	+	-	+	-
Chen, pneumothorax, electromagnetic navigation	-	-	+	+	+	-	+	-
Chen, chest tube insertion (with pneumothorax) , electromagnetic navigation	-	-	+	+	+	-	+	-
Chen, fever, electromagnetic navigation	-	-	+	+	+	-	+	-
Chen, major complication, electromagnetic navigation	-	-	+	+	+	X	+	X
Xu, pneumothorax	-	-	+	+	+	-	+	-
Xu, hemorrhage	-	-	+	+	+	-	+	-
Xu, all	-	-	+	+	+	X	+	X

Domains:
D1: Bias due to confounding.
D2: Bias due to selection of participants.
D3: Bias in classification of interventions.
D4: Bias due to deviations from intended interventions.
D5: Bias due to missing data.
D6: Bias in measurement of outcomes.
D7: Bias in selection of the reported result.

Judgement
X Serious
- Moderate
+ Low
? No information

S7g. Risk of bias – Complications (ROBINS-I)

Study ID	D1	D2	D3	D4	D5	Overall
Teng J. et al.2020	!	+	+	+	!	!
Keshava S.N. et al.2022	!	+	+	+	+	!
Durand P. et al.2017	+	+	+	+	!	!
Heerink W.J. et al.2018	+	+	+	+	!	+
Gruber-Rouh T. et al.2015	!	+	+	+	!	!
Anzidei M. et al.2015	!	+	+	+	!	!
Zhang Z. et al.2018	!	+	+	+	!	!

S7h. Risk of bias – Complications (RoB 2)

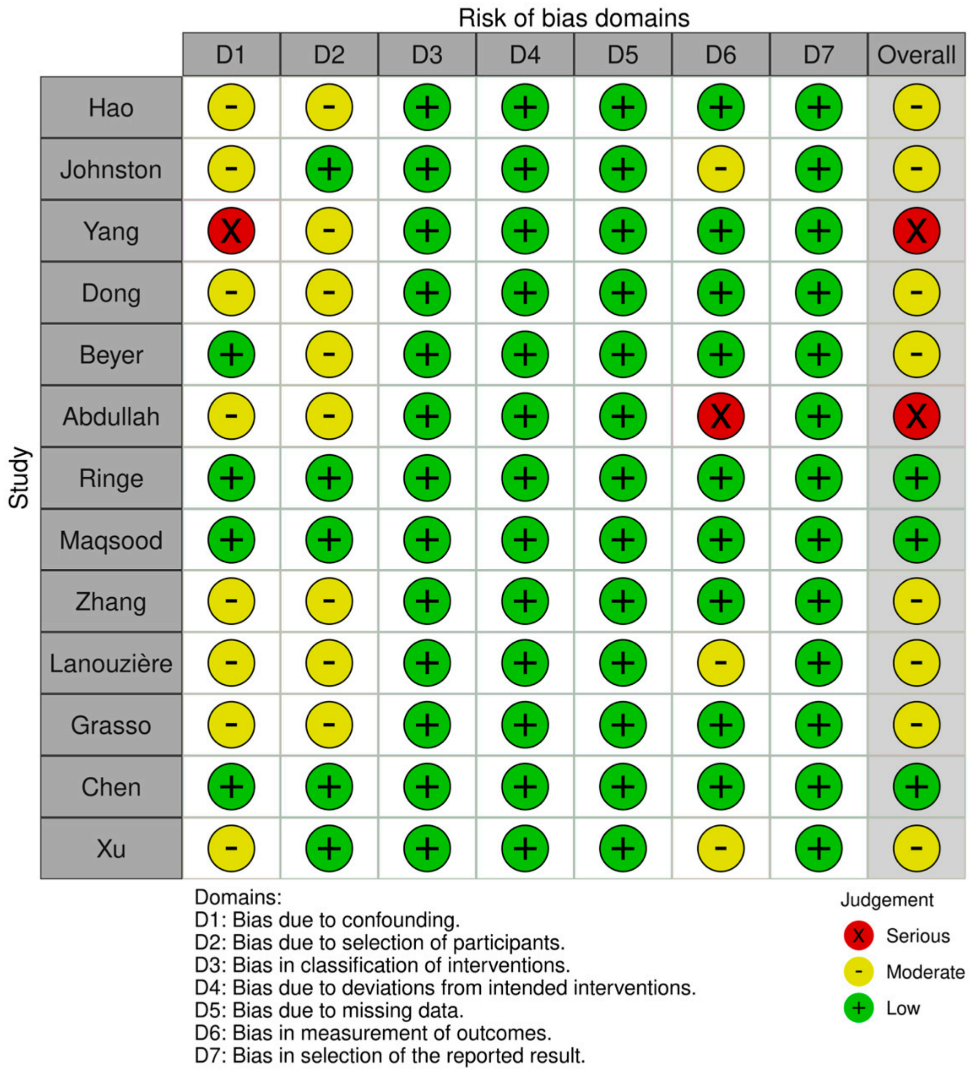

S7i. Risk of bias – Technical success (ROBINS-I)

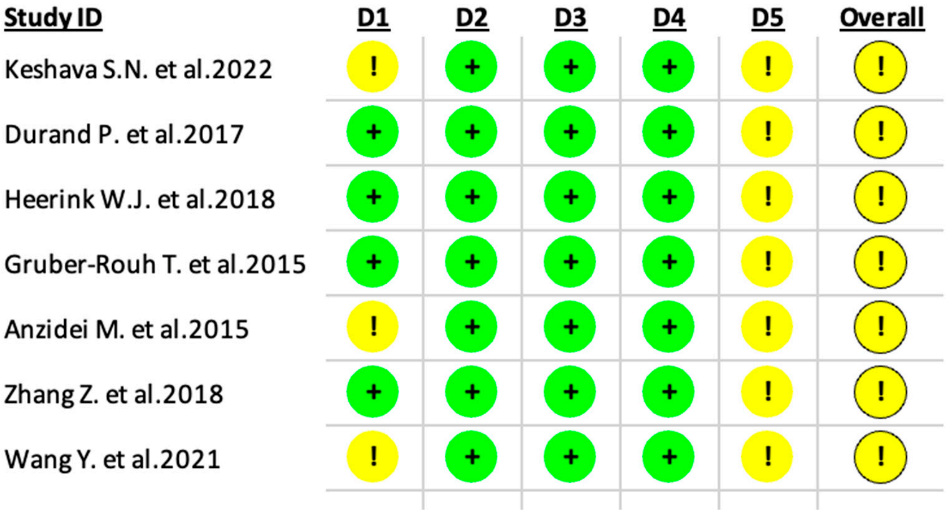

S7j. Risk of bias – Technical success (RoB 2)

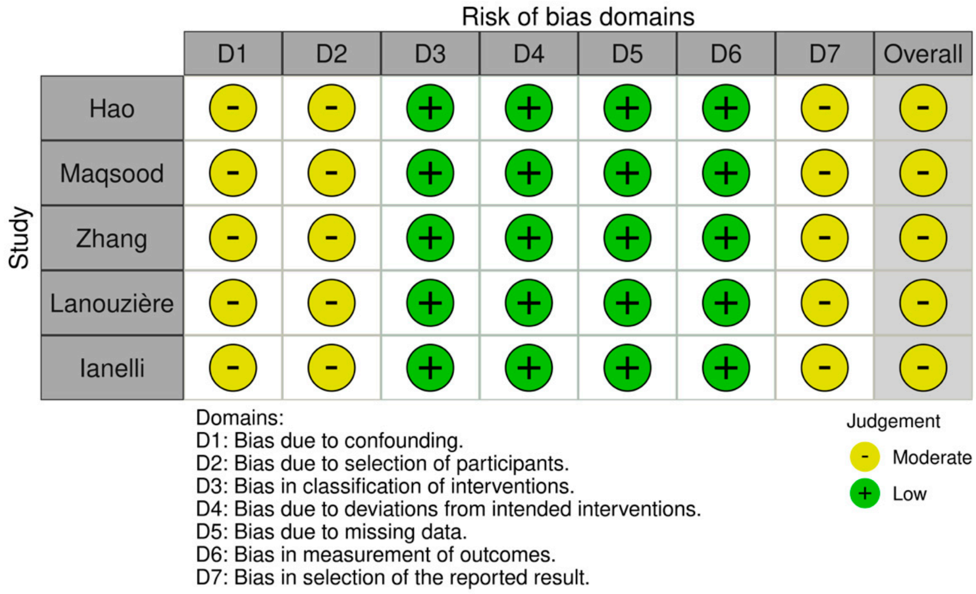

S7k. Risk of bias – Diagnostic success (ROBINS-I)

Study ID	D1	D2	D3	D4	D5	Overall
Liu Q. et al.2023	+	+	+	+	+	+
Anzidei M. et al.2015	+	+	+	+	+	+
Wang Y. et al.2021	!	+	+	+	+	!
Perepelevskiy A.N. et al.2022	!	+	+	+	+	!

S7l. Risk of bias – Diagnostic success (RoB 2)

Supplemental Figure S8. Funnel plots

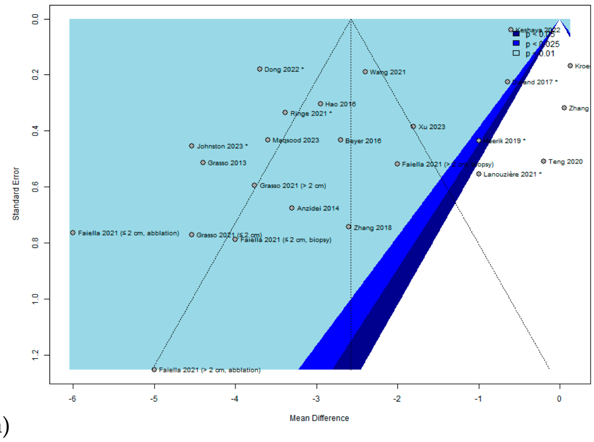

a)

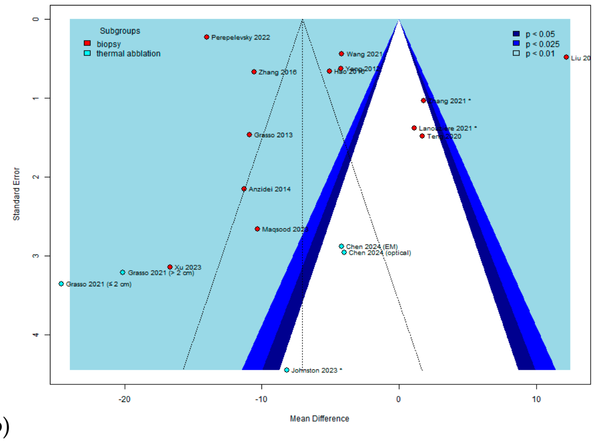

b)

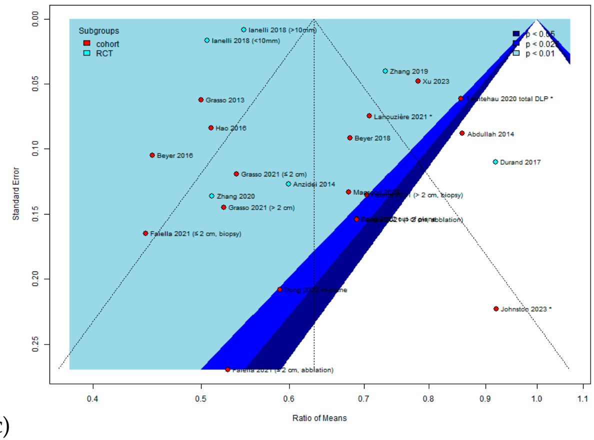

c)

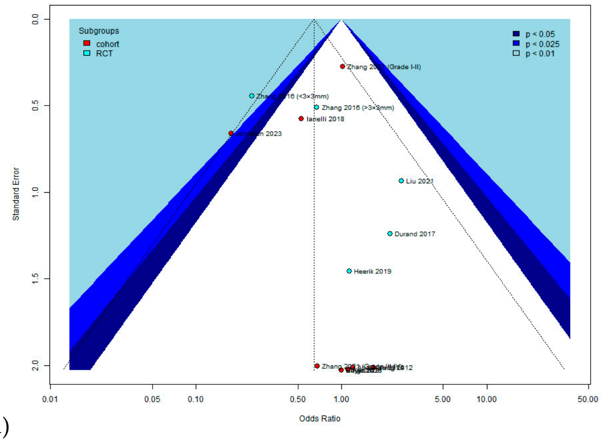

d)

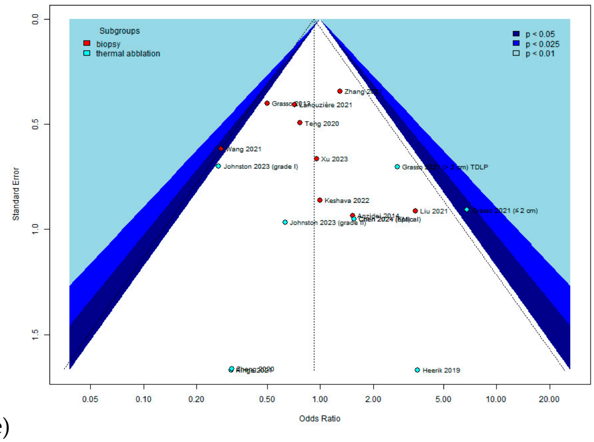

e)

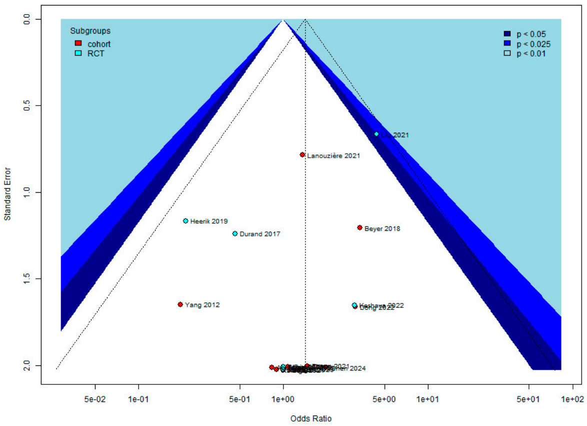

f)

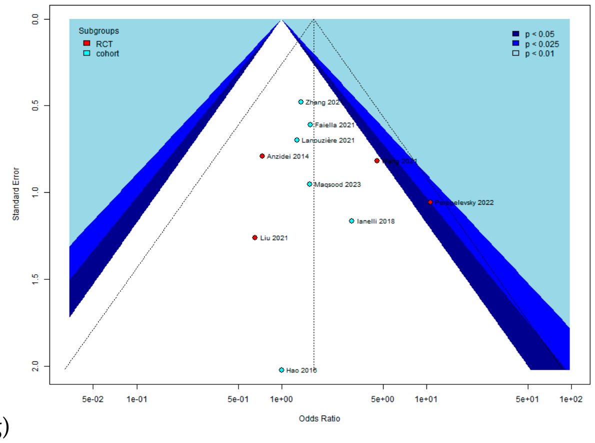

g)

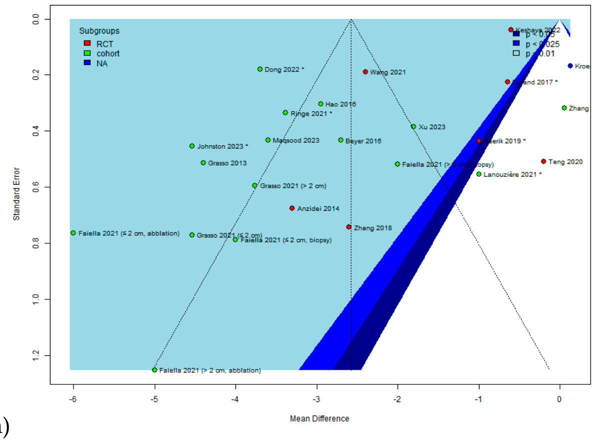

h)

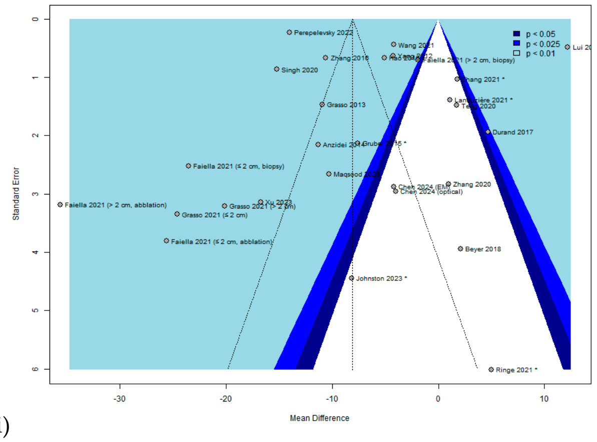

i)

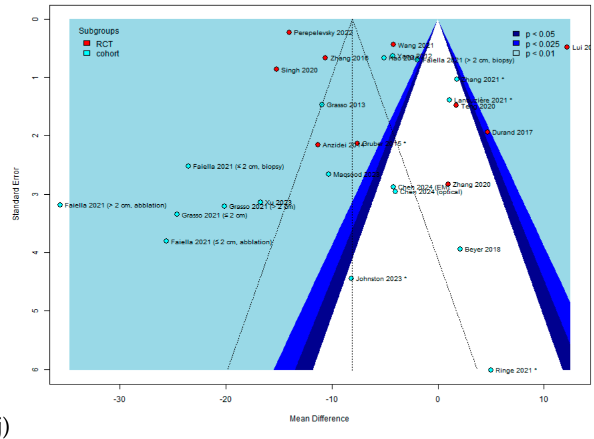

j)

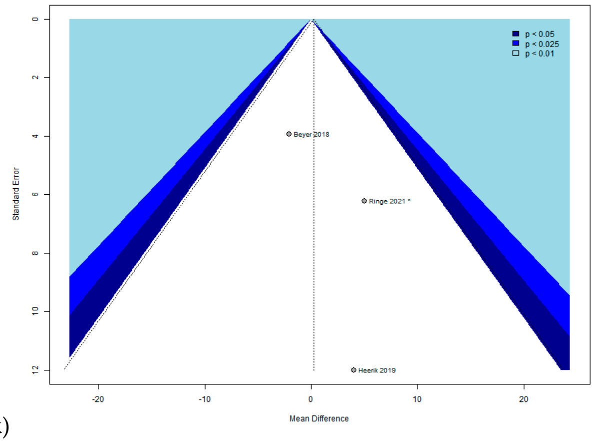

k)

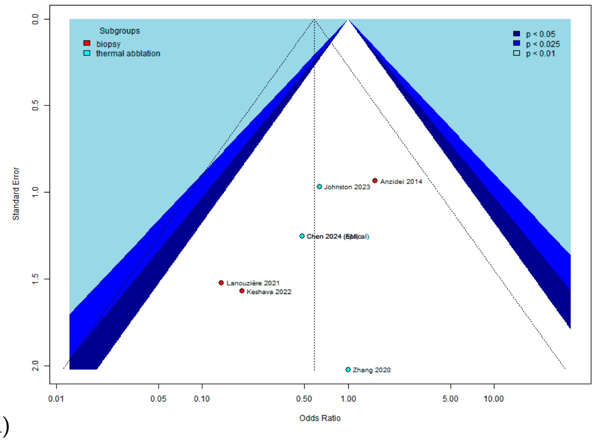

l)

Supplemental Figure S8. Funnel plots for publication bias assessment.

Funnel plots corresponding to the primary and subgroup analyses of each outcome parameter:

a) Needle manipulations (Figure 1)

b) Thoracic procedural time (Figure 2)

c) Irradiation dose (Figure 3)

d) Overall complication rates (Figure 4)

e) Pneumothorax rates (Figure 5)

f) Technical success rates (Figure 6)

g) Diagnostic success rates (Figure 7)

h) Needle manipulations (Supplemental Figure S2)

i) Pooled procedural time (Supplemental Figure S3)

j) Procedural time – subgroup analysis by study type (Supplemental Figure S4)

k) Procedural time for abdominal interventions (Supplemental Figure S5)

l) Chest tube insertion rates in lung thermal ablation and biopsy subgroups (Supplemental Figure S6)
