# Supplementary material for: Navigation Systems Significantly Improve the Efficiency and Safety of CT-Guided Interventions
Source: Life (Basel). 2026 Mar 6;16(3):431. doi: 10.3390/life16030431 (PMC13027737; doi:10.3390/life16030431)
Supplement: Supplementary file 1 [file life-16-00431-s001.zip › Supplemental Methods and Tables.pdf]

Supplementary Methods

Translation of non-English studies

Where required, non-English articles were translated using DeepL (DeepL SE, Cologne, Germany) to ensure accurate data extraction.

Supplemental Tables

Database	MeSH terms	Results
PubMed	(("CT" OR (compute* AND "tomography") OR "image" OR guid* OR “CT-based”) AND ("intervention" OR "ablation" OR "drainage" OR biops* OR “cryobiopsy” OR ("needle" AND ("manipulation" OR "insertion" OR "positioning" OR "placement")) OR "procedure" OR "percutaneous")) AND (navigat* OR "laser" OR "LNS" OR ("augmented" AND "reality") OR "electromagnetic" OR "optical" OR ("robot" AND "assisted") OR "stereotactic" OR “robotic”) AND ("conventional" OR "freehand" OR "free-hand")	2,653 articles
Scopus	(("CT" OR (compute* AND "tomography") OR "image" OR guid* OR “CT-based”) AND ("intervention" OR "ablation" OR "drainage" OR biops* OR “cryobiopsy” OR ("needle" AND ("manipulation" OR "insertion" OR "positioning" OR "placement")) OR "procedure" OR "percutaneous")) AND (navigat* OR "laser" OR "LNS" OR ("augmented" AND "reality") OR "electromagnetic" OR "optical" OR ("robot" AND "assisted") OR "stereotactic" OR “robotic”) AND ("conventional" OR "freehand" OR "free-hand")	9,953 articles
Cohrane Library	(("CT" OR (compute* AND "tomography") OR "image" OR guid* OR “CT-based”) AND ("intervention" OR "ablation" OR "drainage" OR biops* OR “cryobiopsy” OR ("needle" AND ("manipulation" OR "insertion" OR "positioning" OR "placement")) OR "procedure" OR "percutaneous")) AND (navigat* OR "laser" OR "LNS" OR ("augmented" AND "reality") OR "electromagnetic" OR "optical" OR ("robot" AND "assisted") OR "stereotactic" OR “robotic”) AND ("conventional" OR "freehand" OR "free-hand")	1,051 trials
EMBASE	('ct' OR (compute* AND 'tomography') OR 'image' OR guid* OR 'ct-based') AND (('intervention' OR 'ablation' OR 'drainage' OR biops* OR 'cryobiopsy') OR ('needle' AND ('manipulation' OR 'insertion' OR 'positioning' OR 'placement')) OR 'procedure' OR 'percutaneous') AND (navigat* OR 'laser' OR 'lns' OR ('augmented' AND 'reality') OR 'electromagnetic' OR 'optical' OR ('robot' AND 'assisted') OR 'stereotactic' OR 'robotic') AND ('conventional' OR 'freehand' OR 'free-hand')	5,744 articles

Table S1: Search strategies and results across four databases

Section and Topic	Item #	Checklist item	Location where item is reported
**TITLE**			
Title	1	Identify the report as a systematic review.	Title page
**ABSTRACT**			
Abstract	2	See the PRISMA 2020 for Abstracts checklist.	Abstract
**INTRODUCTION**			
Rationale	3	Describe the rationale for the review in the context of existing knowledge.	Introduction
Objectives	4	Provide an explicit statement of the objective(s) or question(s) the review addresses.	Introduction, last paragraph
**METHODS**			
Eligibility criteria	5	Specify the inclusion and exclusion criteria for the review and how studies were grouped for the syntheses.	Materials and Methods – Eligibility
Information sources	6	Specify all databases, registers, websites, organisations, reference lists and other sources searched or consulted to identify studies. Specify the date when each source was last searched or consulted.	Materials and Methods – Information Sources and Search Strategy
Search strategy	7	Present the full search strategies for all databases, registers and websites, including any filters and limits used.	Supplemental Table S1
Selection process	8	Specify the methods used to decide whether a study met the inclusion criteria of the review, including how many reviewers screened each record and each report retrieved, whether they worked independently, and if applicable, details of automation tools used in the process.	Materials and Methods – Study Selection and Data Extraction
Data collection process	9	Specify the methods used to collect data from reports, including how many reviewers collected data from each report, whether they worked independently, any processes for obtaining or confirming data from study investigators, and if applicable, details of automation tools used in the process.	Materials and Methods – Study Selection and Data Extraction
Data items	10a	List and define all outcomes for which data were sought. Specify whether all results that were compatible with each outcome domain in each study were sought (e.g. for all measures, time points, analyses), and if not, the methods used to decide which results to collect.	Materials and Methods – Eligibility Criteria, Data Extraction
	10b	List and define all other variables for which data were sought (e.g. participant and intervention characteristics, funding sources). Describe any assumptions made about any missing or unclear	Materials and Methods –

Section and Topic	Item #	Checklist item	Location where item is reported
		information.	Eligibility Criteria, Data Extraction
Study risk of bias assessment	11	Specify the methods used to assess risk of bias in the included studies, including details of the tool(s) used, how many reviewers assessed each study and whether they worked independently, and if applicable, details of automation tools used in the process.	Materials and Methods – Risk of Bias and Evidence Certainty
Effect measures	12	Specify for each outcome the effect measure(s) (e.g. risk ratio, mean difference) used in the synthesis or presentation of results.	Materials and Methods – Data Synthesis and Statistical Analysis
Synthesis methods	13a	Describe the processes used to decide which studies were eligible for each synthesis (e.g. tabulating the study intervention characteristics and comparing against the planned groups for each synthesis (item #5)).	Materials and Methods – Data Synthesis and Statistical Analysis
	13b	Describe any methods required to prepare the data for presentation or synthesis, such as handling of missing summary statistics, or data conversions.	Materials and Methods – Data Synthesis and Statistical Analysis
	13c	Describe any methods used to tabulate or visually display results of individual studies and syntheses.	Materials and Methods – Data Synthesis and Statistical Analysis
	13d	Describe any methods used to synthesize results and provide a rationale for the choice(s). If meta-analysis was performed, describe the model(s), method(s) to identify the presence and extent of statistical heterogeneity, and software package(s) used.	Materials and Methods – Data Synthesis and Statistical Analysis
	13e	Describe any methods used to explore possible causes of heterogeneity among study results (e.g. subgroup analysis, meta-regression).	Materials and Methods – Data Synthesis and Statistical Analysis
	13f	Describe any sensitivity analyses conducted to assess robustness of the synthesized results.	Materials and Methods – Data Synthesis and Statistical Analysis

Section and Topic	Item #	Checklist item	Location where item is reported
Reporting bias assessment	14	Describe any methods used to assess risk of bias due to missing results in a synthesis (arising from reporting biases).	Materials and Methods – Data Synthesis and Statistical Analysis
Certainty assessment	15	Describe any methods used to assess certainty (or confidence) in the body of evidence for an outcome.	Materials and Methods – Risk of Bias and Evidence Certainty
**RESULTS**			
Study selection	16a	Describe the results of the search and selection process, from the number of records identified in the search to the number of studies included in the review, ideally using a flow diagram.	Results – Study Selection and Characteristics;
	16b	Cite studies that might appear to meet the inclusion criteria, but which were excluded, and explain why they were excluded.	Figure 1
Study characteristics	17	Cite each included study and present its characteristics.	Results – Sections 3.2 to 3.6; Figures 2–6; Supplemental Figures
Risk of bias in studies	18	Present assessments of risk of bias for each included study.	Results – Sections 3.2 to 3.6; Figures 2–6; Supplemental Figures
Results of individual studies	19	For all outcomes, present, for each study: (a) summary statistics for each group (where appropriate) and (b) an effect estimate and its precision (e.g. confidence/credible interval), ideally using structured tables or plots.	Results – Sections 3.2 to 3.6; Figures 2–6; Supplemental Figures
Results of syntheses	20a	For each synthesis, briefly summarise the characteristics and risk of bias among contributing studies.	Results – Sections 3.2 to 3.6; Supplemental Figures
	20b	Present results of all statistical syntheses conducted. If meta-analysis was done, present for each the summary estimate and its precision (e.g. confidence/credible interval) and measures of statistical heterogeneity. If comparing groups, describe the direction of the effect.	forest plots

Section and Topic	Item #	Checklist item	Location where item is reported
	20c	Present results of all investigations of possible causes of heterogeneity among study results.	forest plots
	20d	Present results of all sensitivity analyses conducted to assess the robustness of the synthesized results.	forest plots
Reporting biases	21	Present assessments of risk of bias due to missing results (arising from reporting biases) for each synthesis assessed.	Results – Risk of Bias and GRADE Assessment; Supplemental Figure S9
Certainty of evidence	22	Present assessments of certainty (or confidence) in the body of evidence for each outcome assessed.	Results – Risk of Bias and GRADE Assessment; Supplemental Table S3
**DISCUSSION**			
Discussion	23a	Provide a general interpretation of the results in the context of other evidence.	Discussion, paragraphs 1–5
	23b	Discuss any limitations of the evidence included in the review.	Discussion, paragraphs 6
	23c	Discuss any limitations of the review processes used.	Discussion, paragraphs 6
	23d	Discuss implications of the results for practice, policy, and future research.	Discussion, final paragraph
**OTHER INFORMATION**			
Registration and protocol	24a	Provide registration information for the review, including register name and registration number, or state that the review was not registered.	Materials and Methods – Study Protocol
	24b	Indicate where the review protocol can be accessed, or state that a protocol was not prepared.	Materials and Methods – Study Protocol
	24c	Describe and explain any amendments to information provided at registration or in the protocol.	Materials and Methods – Study Protocol
Support	25	Describe sources of financial or non-financial support for the review, and the role of the funders or	Abbreviated Title

Section and Topic	Item #	Checklist item	Location where item is reported
		sponsors in the review.	Page
Competing interests	26	Declare any competing interests of review authors.	Abbreviated Title Page
Availability of data, code and other materials	27	Report which of the following are publicly available and where they can be found: template data collection forms; data extracted from included studies; data used for all analyses; analytic code; any other materials used in the review.	Materials and Methods – Reporting Standards

Table S2: PRISMA checklist

Table S3: GRADE Summary of Findings

Navigation systems compared to freehand method for patients undergoing CT guided intervention

Certainty assessment							Summary of findings				
							Study event rates (%)			Anticipated absolute effects	
Participants (studies) Follow-up	Risk of bias	Inconsistency	Indirectness	Imprecision	Publication bias	Overall certainty of evidence	With freehand method	With navigation systems	Relative effect (95% CI)	Risk with freehand method	Risk difference with navigation systems
**Number of needle manipulations - RCTs (assessed with: Reported needle manipulation count, or control CT count. ; Scale from: 0 to 6)**											
685 (10 non-randomised studies)	not serious	serious	not serious	not serious	none	⨁⨁⨁◯ Moderate	342	343	-	342	**0** (0 to 0)
**Number of needle manipulations - cohort studies (assessed with: Reported needle manipulation count, or control CT count. ; Scale from: 0 to 9)**											
1211 (12 non-randomised studies)	not serious	serious[a]	not serious	not serious	none	⨁⨁⨁◯ Moderate[a]	517	694	-	517	**0** (0 to 0)
**Procedural time - RCTs**											

Table S3: GRADE Summary of Findings

Navigation systems compared to freehand method for patients undergoing CT guided intervention

**Certainty assessment**							**Summary of findings**				
1137 (11 RCTs)	not serious	serious[b]	not serious	not serious	none	⊕⊕⊕◯ Moderate[b]	568	569	-	568	**0** (0 to 0)
**Procedural time - cohort studies**											
1449 (15 non-randomised studies)	not serious	serious[b]	not serious	not serious	none	⊕⊕⊕◯ Moderate[b]	656	793	-	656	**0** (0 to 0)
**Irradiation dose - RCTs**											
643 (5 RCTs)	serious[c]	not serious	not serious	not serious	none	⊕⊕⊕◯ Moderate[c]	323	320	-	323	**0** (0 to 0)
**Irradiation dose - cohort**											
1101 (14 non-randomised studies)	not serious	not serious	not serious	not serious	none	⊕⊕⊕⊕ High	483	618	-	483	**0** (0 to 0)
**Technical success rate - cohort srtudies**											

Table S3: GRADE Summary of Findings

Navigation systems compared to freehand method for patients undergoing CT guided intervention

Certainty assessment							Summary of findings				
988 (13 non-randomised studies)	not serious	not serious	not serious	not serious	none	⊕⊕⊕⊕ High	464/472 (98.3%)	511/516 (99.0%)	not estimable	464/472 (98.3%)	

Technical success rate - RCT

502 (8 RCTs)	not serious	not serious	not serious	not serious	none	⊕⊕⊕⊕ High	236/249 (94.8%)	242/253 (95.7%)	not estimable	236/249 (94.8%)	

Diagnostic success rate - cohort studies

657 (5 non-randomised studies)	not serious	not serious	not serious	not serious	none	⊕⊕⊕⊕ High	334286/306 (109243.8%)	334/351 (95.2%)	not estimable	334286/306 (109243.8%)	

Diagnostic success rate - RCT

543 (5 RCTs)	not serious	not serious	not serious	not serious	none	⊕⊕⊕⊕ High	246/268 (91.8%)	266/275 (96.7%)	not estimable	246/268 (91.8%)	

Complication rates - cohort studies

Table S3: GRADE Summary of Findings

Navigation systems compared to freehand method for patients undergoing CT guided intervention

Certainty assessment							Summary of findings					
994 (8 non-randomised studies)	not serious	serious[d]	serious[e]	not serious	none	⊕⊕◯◯ Low[d,e]	59/467 (12.6%)	63/527 (12.0%)	not estimable	59/467 (12.6%)		
**Complication rates - RCTs**												
397 (4 RCTs)	not serious	serious[f]	serious[f]	not serious	none	⊕⊕◯◯ Low[f]	41/202 (20.3%)	25/195 (12.8%)	not estimable	41/202 (20.3%)		

CI: confidence interval

Explanations

a. Very high heterogeneity ($I^2 = 97\%$), suggesting variability in true effects likely due to different operator experience, lesion location, and type of navigation system used.

b. Extremely high heterogeneity ($I^2 = 99\%$), possibly due to procedural type, operator familiarity with NS, and setup time differences.

c. One major outlier (Heerink) showed skewed results due to longer scan lengths, affecting pooled estimates.

d. Moderate heterogeneity ($I^2 = \sim 50\%$), likely from different definitions and severities of complications.

e. Variation in reported types of complications (e.g., pneumothorax, hemorrhage, chest tube insertion) makes pooling more complex.

f. Definitions of complications were inconsistent, and event rates were low, widening the confidence intervals.
